# GPRC5C regulates the composition of cilia in the olfactory system

**DOI:** 10.1186/s12915-023-01790-0

**Published:** 2023-12-18

**Authors:** Sneha Bhat, André Dietz, Katja Senf, Sandor Nietzsche, Yoshio Hirabayashi, Martin Westermann, Eva Maria Neuhaus

**Affiliations:** 1 Pharmacology and Toxicology, Jena University Hospital, Friedrich Schiller University Jena, Drackendorfer Str. 1, 07747 Jena, Germany; 2 Centre for Electron Microscopy, Jena University Hospital, Friedrich Schiller University Jena, Ziegelmühlenweg 1, 07743 Jena, Germany; 3 Institute for Environmental and Gender-Specific Medicine, Juntendo University Graduate School of Medicine, Chiba, 279-0021 Japan; 4 RIKEN Cluster for Pioneering Research, RIKEN, Wako, Saitama 351-0198 Japan

**Keywords:** GPRC5C, Olfactory, Sensory, Cilia, Smell

## Abstract

**Background:**

Olfactory sensory neurons detect odourants via multiple long cilia that protrude from their dendritic endings. The G protein-coupled receptor GPRC5C was identified as part of the olfactory ciliary membrane proteome, but its function and localization is unknown.

**Results:**

High-resolution confocal and electron microscopy revealed that GPRC5C is located at the base of sensory cilia in olfactory neurons, but not in primary cilia of immature neurons or stem cells. Additionally, GPRC5C localization in sensory cilia parallels cilia formation and follows the formation of the basal body. In closer examination, GPRC5C was found in the ciliary transition zone. GPRC5C deficiency altered the structure of sensory cilia and increased ciliary layer thickness. However, primary cilia were unaffected. Olfactory sensory neurons from *Gprc5c*-deficient mice exhibited altered localization of olfactory signalling cascade proteins, and of ciliary phosphatidylinositol-4,5-bisphosphat. Sensory neurons also exhibited increased neuronal activity as well as altered mitochondrial morphology, and knockout mice had an improved ability to detect food pellets based on smell.

**Conclusions:**

Our study shows that GPRC5C regulates olfactory cilia composition and length, thereby controlling odour perception.

**Supplementary Information:**

The online version contains supplementary material available at 10.1186/s12915-023-01790-0.

## Background

The murine olfactory epithelium (OE) can sense a myriad of chemosensory stimuli, which are essential for almost every aspect of life. The odourants are detected by bipolar olfactory sensory neurons (OSNs) in the nasal cavity [1]. Apical dendrites of mature OSNs end in a dendritic knob, from where 20–30 cilia protrude into the thick mucosal layer covering the surface of the epithelium. The cilia in terminally differentiated OSN are remarkably different from primary cilia in most tissues [2, 3]. Although olfactory cilia have a (9 × 2 + 2) microtubule configuration normally found in motile cilia, they are immotile due to a lack of the dynein arms necessary for movement [4]. With lengths between 2.5 and 110 µm [5], olfactory cilia are optimized for detecting external stimuli by the olfactory signal transduction cascade components. Signalling starts with ligand binding to odourant receptors, which triggers the olfactory‐specific G protein G_αolf_ (GNAL) [6] to activate adenylyl cyclase type III (ADCY3, AC3) [7], enabling cAMP-driven opening of cyclic nucleotide gated channels (CNG) [8] and depolarization. Anosmia is caused by disruption of cilia function and structure, which is evidence of the necessity of cilia as special membrane area for odourant detection [3, 9, 10]. There is emerging evidence that the lipid composition of the ciliary membrane also varies from that of the olfactory neuron plasma membrane, and modulates the odour response [11, 12].

Similar to olfactory cilia, non-motile primary cilia act as a sensors for extracellular signals [13]. All cilia lack the machinery for synthesizing proteins required for signalling and components necessary for its own formation, functioning and maintenance. Assembly and disassembly of cilia therefore requires selective import/export of cilium-specific proteins through a diffusion barrier at the ciliary base. This ciliary gate at the juncture between the plasma membrane and the ciliary membrane comprises the transition fibres (TFs) and the transition zone (TZ) [14, 15]. TFs are the distal appendages of the basal body that anchor it to the ciliary membrane. The TZ is located above the TFs and extends into the proximal segment of the cilium, consisting of membranous particles that form characteristic Y-links to connect the microtubules to the ciliary membrane [15]. In freeze-fracture studies, the TZ appears as rows of bead-like particles spanning the circumference of the ciliary membrane in the proximal segment, referred to as the ciliary necklace [16]. The TZ functionally integrates ciliary trafficking systems that cross the barrier and constitute the ciliary compartment. Entry and exit of membrane proteins into cilia employs intraflagellar transport (IFT) cargo adaptor complexes and Bardet–Biedl syndrome (BBS) proteins [17, 18], whereas targeting of prenylated and myristoylated proteins requires solubilization of the cargo and the release by the ciliary small G-protein ARL3 [19]. Mutations in genes that disrupt IFT, BBSome or any of the proteins at the ciliary gate manifest in a group of diseases collectively termed as ciliopathies, which arise due to cilia dysfunction with varying level of penetrance in different ciliated tissues [20]. Ciliopathies are often characterized by developmental defects with phenotypes ranging from renal anomalies, congenital heart defects, hydrocephalus, obesity, infertility to hearing loss, and anosmia [20].

Although many ciliopathies result in anosmia [21–23], OSN cilia are built and maintained differently from primary cilia. For example, the BBS protein BBS4 is required for ciliary localization of G protein-coupled receptors in neurons of the central nervous system [24], but not for ciliary localization of odourant receptors [21], suggesting that olfactory cilia have a unique mechanism in regulating ciliary protein transport. The composition of the transition zone, which can differ between cilia types within one organism [25, 26], remains largely unknown in olfactory cilia. Loss of some well-known transition zone proteins (CEP290, MKS1 and MKS3) shows an olfactory phenotype [9, 27]. On the other hand, deletion of *Cc2d2a* (MKS6), a core transition zone component, in all tissues of postnatal mice results in kidney and retinal phenotypes, but does not result in anosmia [28]. Moreover, olfactory cilia typically have more strands of intramembrane particles in their ciliary necklace than primary cilia or respiratory cilia [29]. The organization, composition and function of the transition zone in olfactory cilia is not fully elucidated yet.

We found previously that *Gprc5c* mRNA was abundantly expressed in the OE and GPRC5C was co-purified with olfactory cilia [30, 31]. To address the contribution of GPRC5C towards the function of OSN cilia, we investigated here the OE of a *Gprc5c* knock-out mouse model. GPRC5 receptors A–D constitute an unusual group of chordate-specific class C G protein-coupled receptors (GPCRs), since the receptors share class A GPCR topology with short N and C-termini [32, 33]. G protein-coupled receptor, family C, group 5, member C (GPRC5C) appears to be expressed in different tissues [34]. *Gprc5c*-deficient (*Gprc5c*^−/−^) mice are viable and fertile and showed no significantly abnormal behaviour in several tests, except for signs of alterations of the hematopoetic system [35]. GPRC5C was described to take part in the regulation of NHE3, the apical membrane of renal proximal tubules, and to be activated by alkaline pH [36]. In another study, GPRC5C was found to regulate dormancy of haematopoetic stem cells upon activation by N-acetylglucosamine and hyaluronic acid [37]. Here we show that GPRC5C regulates cilia length and composition and thereby alters the odour response of OSNs.

## Methods

### Mice

Animal experiments were conducted in accordance with the EC directive 86/609/European Economic Community guidelines for animal experiments and permitted by the local government (Thüringer Landesamt für Lebensmittelsicherheit und Verbraucherschutz). Mice were kept under 12 h light/dark cycles with ad libitum access to food and water. C57BL6/6 J wild type mice were originally purchased from Charles River Laboratories (Sulzfeld, GER) *Gprc5c knockout* (Gprc5c^tm1Yhir^, Gprc5c^−/−^) mice with C57BL/6 background [35] were transferred from Nina Cabezas-Wallscheid (Department of Stem Cells and Cancer, German Cancer Research Center (DKFZ); Heidelberg Institute for Stem Cell Technology and Experimental Medicine). Genotyping primer sequences and conditions were used as described [35]. For regeneration experiments, mice at an age of 2 M were treated intraperitoneally with 50 mg/kg methimazole (Sigma-Aldrich, St. Louis, MO) or 0.9% sodium chloride as a control. Mice were euthanized with an overdose of isoflurane and decapitated.

### Quantitative real-time PCR (qPCR)

The OE lining the septum and in the turbinates, OB, cerebellum, rest brain, spinal cord, tongue, liver, kidney, pancreas, stomach, lung, spleen, and skeletal muscle were collected. Total RNA was isolated using the Purelink RNA Mini Kit (Thermo Fisher Scientific Germany Ltd. & Co. KG, Bonn, GER). cDNA was prepared using the High Capacity cDNA Kit (Thermo Fisher Scientific Germany Ltd. & Co. KG, Bonn, GER). qPCR was performed on a Quant Studio® 3 Real Time PCR Cycler (Thermo Fisher Scientific Germany Ltd. & Co. KG, Bonn, GER) using Power Up SYBR® Green Master Mix (Thermo Fisher Scientific Germany Ltd. & Co. KG, Bonn, GER). Predesigned Quantitect primers for GAPDH (QT01658692) and GPRC5C (QT00148582) were used (Qiagen N.V., Hilden, GER). At least 3 animals were used per experimental group and 3 independent runs were performed. PCR conditions were as follows: 2 min 50 °C, 2 min 95 °C, 15 s 95 °C, 1 min 55 °C, 15 s 95 °C, 1 min 60 °C, 15 s 95 °C for 44 cycles. mRNA Expression levels were calculated using the ddCt method [38].

### *Fluorescence *in situ* hybridization (FISH)*

To study the cellular localization of *Gprc5c* in the OE, FISH was performed as described [39]. The OE was frozen in 2-Methyl Butane at approximately − 25 °C and embedded in Tissue Freezing Medium (Leica Biosystems, 14,020,108,926). Coronal sections of 20-µm thickness were made using a Leica CM3050S Cryostat (Leica Biosystems, Wetzlar, GER) and collected onto SuperFrost Plus™ cryoslides (Thermo Fisher Scientific Germany Ltd. & Co. KG, Bonn, GER). pcDNA3.1 containing *Gprc5c* (corresponding to bp 67–1389 from *Gprc5c*, NM_001110338.1) was used for the synthesis of the sense (negative) and antisense (positive) probes, respectively. Hybridized slides were incubated in anti-digoxigenin-alkaline phosphatase (AP), Fab fragments (Roche Deutschland Holding GmbH, Grenzach-Wyhlen, GER) (1:500), washed and incubated in TSA™ Plus Cyanine 3 (Perkin Elmer, MA, USA). The slides were mounted with coverslips using Fluoromount-G™.

### (Immuno-)histochemistry

Tissue was fixed in 4% PFA solution overnight at 4 °C, transferred to 30% sucrose solution for cryopotection, frozen, and embedded in Tissue Freezing Medium (Leica Biosystems, 14,020,108,926). Coronal sections of 16-µm thickness were made using a Leica CM3050S Cryostat (Leica Biosystems, Wetzlar, GER) and collected onto SuperFrost Plus™ cryoslides (Thermo Fisher Scientific Germany Ltd. & Co. KG, Bonn, GER). Antigen retrieval was performed with citrate buffer (0.1 mol/l Tri-sodiumcitrate dehydrate, 0.5% Tween 20, pH 6) at 97–99 °C in a steamer (Tefal Vitacuisine, Sarcelles, FRA). The sections were incubated in blocking buffer (2% BSA, 3% Normal Donkey Serum, 0.1% Triton in TBS) with antibody solutions (Additional file 1: Table S1) and mounted with coverslips using Fluoromount-G™. For en face labelling, the tissue was fixed in 4% PFA for 15 min, the OE was separated from the septum by gently lifting it off with a pair of fine tweezers and transferred onto a Polysine adhesion slide (Thermo Fisher Scientific Germany Ltd. & Co. KG, Bonn, GER; J2800AMNZ) such that the ciliary side was on top. X-Gal Staining was performed with a kit (Roche Deutschland Holding GmbH, Grenzach-Wyhlen, GER) according to the manufacturer’s instructions.

### Microscopy and quantification

Confocal laser scanning images were produced with either the TCS SPE (Leica DM2500, Leica Microsystems, Wetzlar, GER) or Zeiss LSM 900 with Airyscan 2 (Carl Zeiss, Oberkochen, GER) microscopes. Images were further processed with LAS AF (Leica Microsystems) or Zen (Zeiss) software, ImageJ, and Photoshop CS6 (Adobe Systems, CA, USA). Overview images were taken with a fluorescence stereo microscope M205FA (Leica Microsystems). Bright-field images for the ß-Gal staining were made with a light microscope, Zeiss Axio Imager A1 (Carl Zeiss Oberkochen, GER). For most of the stainings, integrated density measurements were made. Per animal, 4 regions of interest, i.e., 2 on the septum, 1 each from the endo- and ecto-turbinate were imaged and 3–4 animals were used per genotype. Integrated density calculation allows for measuring staining intensity normalized to the area of the region of interest with ImageJ. The image was converted to a 16-bit greyscale image, and the scale was set. A background measurement was taken, followed by measurement of fluorescent particles.

### Western blotting

Olfactory epithelium from four adult mice per genotype was collected and the protein concentration of the samples was measured. Equal amounts of solubilized protein samples were mixed with Laemmli buffer, separated in SDS gels and transferred to nitrocellulose membrane. Membranes were blocked (50 mM NaCl, 150 mM Tris, 0.1% Tween‐20, 5% dry milk, pH 7.4), incubated with the antibodies, and imaged on a Western blot imager (FusionFX, Vilber, France) using ECL Select™. Blots were reprobed with actin antibody to confirm equal loading.

### Methimazole treatment

Methimazole (1-Methyl-2-imidazolethiol, MMZ) is an olfactory toxicant in rodents [40, 41]. C57BL6/6 J WT mice were injected intraperitoneally, either with MMZ (50 mg/kg) (Sigma-Aldrich, St. Louis, MO, USA; 8506) or with 0.9% NaCl as a control. The mice were then euthanized with an overdose of isoflurane 3-, 14- or 28-days post-injection (dpi).

### Freeze fracture immunogold labelling (FRIL)

The OE was separated from the septum, cooled in liquid nitrogen, mounted on copper sandwich profiles and rapidly frozen in a 1:1 ethane/propane mixture, cooled by liquid nitrogen. Tissue pieces were mounted on a brass table that was inserted into the freeze fracture unit (BAL-TEC, BAF400T, LIE). The tissue was fractured at − 150 °C, and replica of the fractured surface samples were made by evaporating a fine layer of platinum–carbon (1.5 nm) at an angle of 35° onto the specimen followed by strengthening the replica by evaporating a layer of electron lucent carbon (15–20 nm) from perpendicular. The replica were directly mounted on grids and used for TEM (EM902a, Zeiss, Oberkochen, GER). TEM images were recorded with a 1 k FastScan-CCD-camera 1024 × 1024 pixels (TVIPS, Munich, GER).

For freeze-fracture immunogold labelling [42], replica were cleaned overnight in SDS buffer (10 mM Tris pH 8.3, 2.5% SDS, 30 mM sucrose), blocked for 30 min in labelling blocking buffer (1% BSA, 0.5% fish gelatin, 0.005% Tween 20 in PBS), and incubated with antibody solutions, and secondary antibody coupled to gold particles (BBI Solutions, Cramlin, UK) were used. The antibody labelling was fixed by incubating the replica in 0.5% glutaraldehyde (Sigma-Aldrich, St. Louis, MO, USA) in PBS for 10 min.

### Transmission electron microscopy

OE from the septum was fixed with 4% PFA and 2.5% glutaraldehyde in cacodylic buffer (0.1 M sodium cacodylate, pH 7.2) for 2 h at RT and 4 °C overnight. The tissue was stained with 1% osmium tetroxide for 1 h, dehydrated with increasing ethanol concentrations (30, 50, 70, 80, 90 and 100%) accompanied by Uranyl acetate staining at 50% ethanol, followed by infiltration of the tissue with graded propylene oxide/epoxy resin (Araldite) series (2:1, 1:1, 1:2). The tissue was embedded in moulds with epoxy resin and cured for 72 h at 60 °C. The embedded tissue was trimmed, ultra-thin (60 nm) sectioned using a Leica Ultracut S (Leica, Wetzlar, Germany), and stained with lead citrate. Finally, the specimens were studied in a transmission electron microscope (EM 900, Zeiss, Oberkochen, Germany) at 80 kV and a magnification of × 20,000. Imaging was done by exposing negatives.

### Scanning electron microscopy (SEM)

OE was fixed as described in the previous section. After washing, the samples were dehydrated in ascending ethanol concentrations (30, 50, 70, 90 and 100%) for 15 min each. Subsequently, the samples were critical-point dried via liquid CO_2_ using a CPD300 (Leica GmbH, Wetzlar, Germany) and sputter coated with gold (thickness approx. 2 nm) using a CCU-010 sputter coater (Safematic GmbH, Zizers, Switzerland). The specimens were investigated with a field emission SEM LEO-1530 Gemini (Carl Zeiss NTS GmbH, Oberkochen, Germany).

### Buried food test

For the buried food test or the cookie finding test [43], mice were fasted 18 h before the test, water however was provided ad libitum. Mice were introduced into a clean cage with bedding material, in which a piece of Froot Loops® (Kellogs) was hidden. The latency to find the buried food was recorded, after 15 min the test was stopped. WT and *Gprc5c*^−/−^ mice were used at an age of 6 weeks (*n* = 20/genotype, total *n* = 40).

### Quantification and statistical analysis

ImageJ was used for quantification and all statistical analysis was made using the GraphPad Prism 6.01. Data were tested for normal distribution and homogeneity in order to check the requirements for parametric tests. All data represent mean ± SEM.

## Results

### GPRC5C is expressed in OSN

**Fig. 1:**
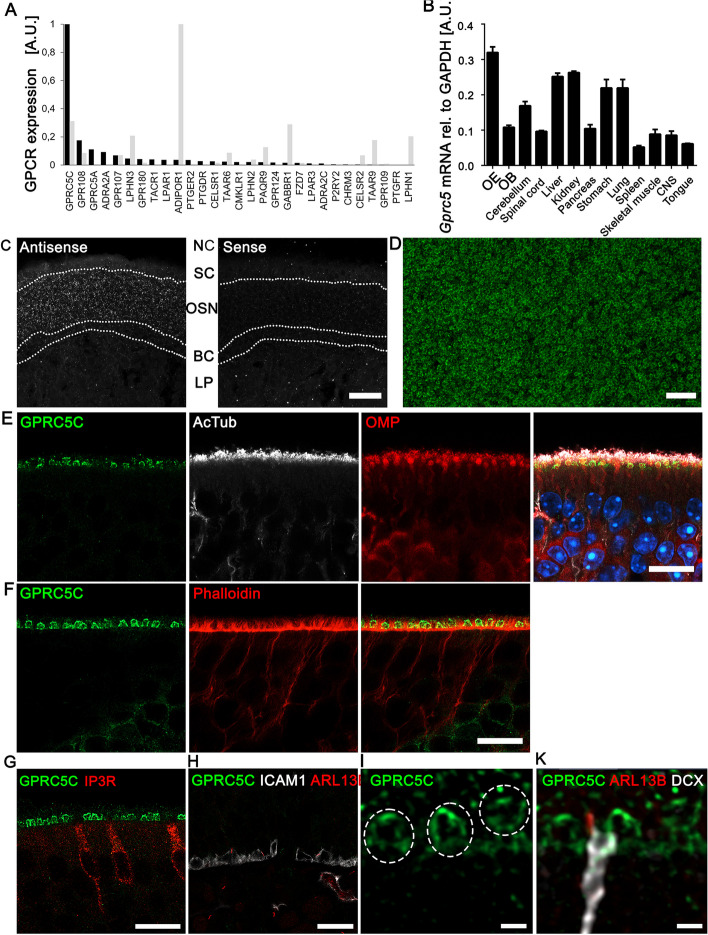
GPRC5C is expressed in dendritic knobs of OSNs. **A** Relative expression levels of GPCRs in cilia preparations of OSNs (black bars) and in next-generation sequencing results of FACS sorted OSNs (grey bars), shown are relative intensity or FPKM levels, each normalized to the most abundant GPCR in both data sets. Shown are non-olfactory GPCRs that have been identified with proteomic methods. **B** qPCR analysis of *Gprc5c* mRNA expression across different tissues in adult mice. CNS is brain excluding olfactory bulb (OB) (*n* = 3, individual data values Additional file 2).** C** Confocal images of adult OE cryosection upon FISH of DIG-labelled probes for GPRC5C (antisense and sense). Strong GPRC5C mRNA expression is detected in the neuronal cell layer (OSN). The apical sustentacular cell layer (SC) and basal cell layers (BC) do not express GPRC5C mRNA. Dotted lines demarcate the different cell layers. NC: Nasal cavity, LP: Lamina propria. Sense control showed no labelling. Scale bar: 20 µm.** D** En face preparation of the OE showing expression of GPRC5C in all dendritic knobs. Confocal images of adult OE coronal cryosection immunolabelled for** E** GPRC5C (green), acetylated-tubulin (AcTub) (white), OMP (red), and overlay along with DAPI (blue) showing localization in dendritic knobs of olfactory neuron **F** GPRC5C (green) and Phalloidin (red) showing GPRC5C + structures embedded in between sustentacular cell microvilli.** G** GPRC5C (green) and IP3R (red) showing that GPRC5C is not localized to the apical surface of microvillar cells. **H** Absence of GPRC5C (labelled in green) in ICAM-positive (white) HBCs, primary cilia are labelled with ARL13B (red), white line marks the basal lamina. **I** GPRC5C (green)-positive dendritic knobs, single-channel image for overlay shown in **K**. **K** ARL13B (red)-positive primary cilia are present on immature OSNs labelled with DCX (white), but not on GPRC5C (green) labelled dendritic knobs. Scale bars **E–H** 10 µm and **I–K** 1 µm

The olfactory epithelium is an interesting model system for the study of cilia dysfunction [3]. We previously analysed the ciliary proteome of OSNs [30] and identified well-known and so far uncharacterized membrane proteins, among them GPRC5C as the most abundant G protein-coupled receptor (Fig. 1A). Next-generation sequencing of purified OSNs confirmed the proteomic results [31] and showed that *Gprc5c* is one of the most abundant receptor transcript in FACS-sorted OSNs (Fig. 1A). Quantitative PCR analysis showed that *Gprc5c* mRNA is expressed in different tissues, along with a prominent expression in the OE (Fig. 1B). Fluorescence in situ hybridization showed *Gprc5c* mRNA localized exclusively in the middle region of the OE, corresponding to the OSN layer (Fig. 1C).

Generation of a specific antibody allowed localization of GPRC5C in the dendritic knobs, to the base of the olfactory cilia in all or nearly all neurons (Additional file 1: Fig. S1; Fig. 1D). In order to confirm the localization of GPRC5C in dendritic knobs, we performed immunofluorescence labelling along with marker proteins corresponding to different cell types of the OE. Co-immunostaining of GPRC5C with acetylated-tubulin, marker for olfactory cilia and olfactory marker protein (OMP), a marker for mature OSN, revealed an intense labelling of GPRC5C in dendritic knobs of OSN, directly beneath the layer of enmeshed olfactory cilia (Fig. 1E). GPRC5C could also be detected in dendritic knobs of immature GAP43-positive OSN (Additional file 1: Fig. S2). Concomitant with the increase in the number of mature OSNs during postnatal development, a significant increase in the number of knobs expressing GPRC5C was observed, indicating that GPRC5C expression is relevant in mature OSN (Additional file 1: Fig. S2A-D).

Co-labelling of GPRC5C with fluorophore conjugated phalloidin shows GPRC5C staining in between, but not overlapping with the microvilli of sustentacular cells (Fig. 1F). Moreover, GPRC5C labelling was absent from the apical surface of IP3R-positive microvillar cells [44], proposed to be play a role in viral infection defense of the OE (Fig. 1G). Since we observed the localization of GPRC5C near the base of cilia in OSNs, we investigated its association with primary cilia in horizontal basal cells, known to possess primary cilia as sensors of growth signals and differentiation cues [45]. Co-labelling of GPRC5C with ICAM as a marker for HBCs and with the primary ciliary marker ARL13B revealed an absence of GPRC5C staining in horizontal basal cells (Fig. 1H). In addition, GPRC5C was not clearly localized to primary cilia in proximal tubules of the kidney (Additional file 1: Fig. S2E) or in doublecortin (DCX)-positive immature OSN (Fig. 1I, K). Thus, GPRC5C localizes to the base of olfactory cilia in the dendritic knobs of OSNs, but not to all primary cilia.

### GPRC5C expression in the developing and regenerating OE

**Fig. 2:**
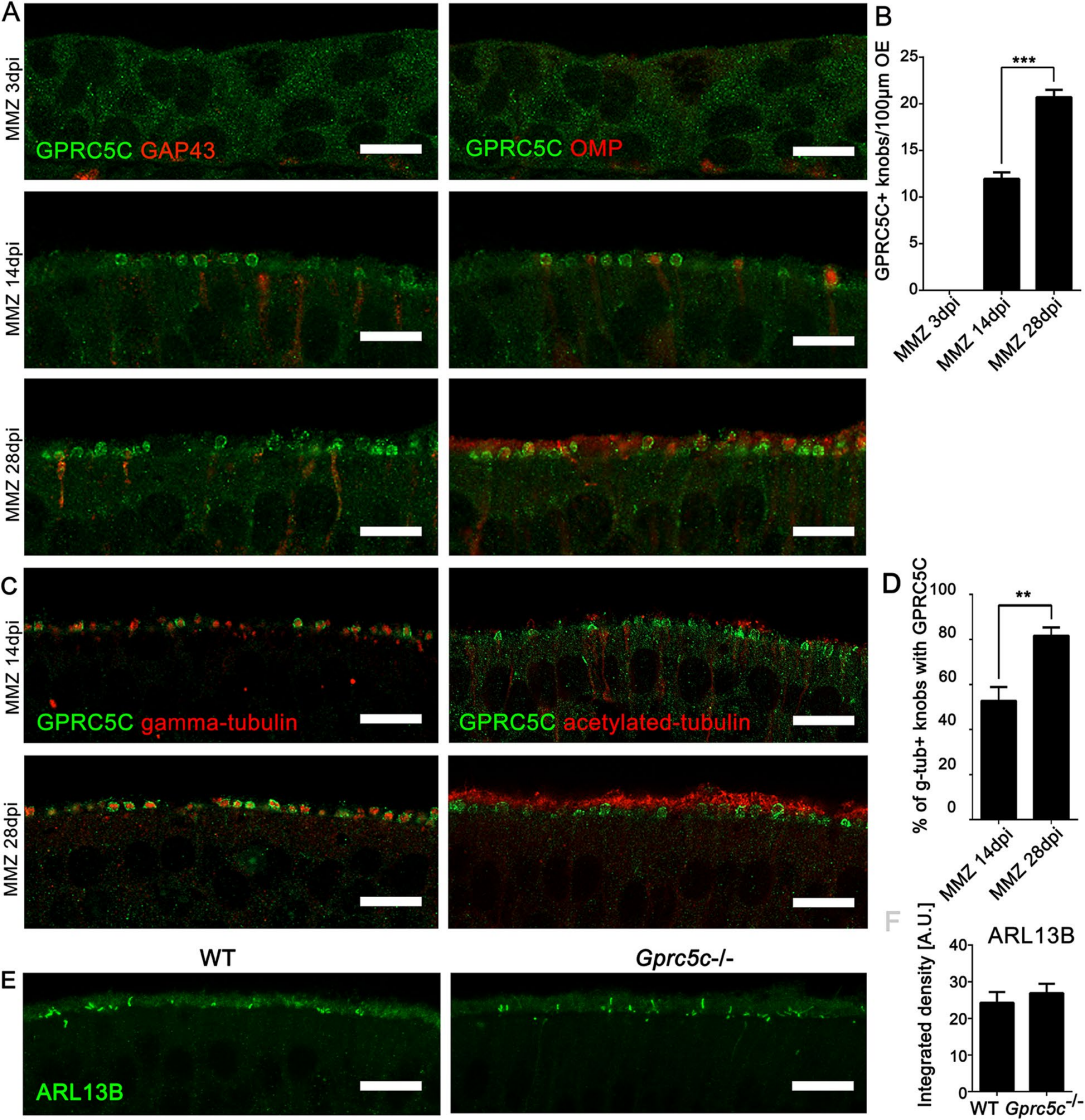
Localization of GPRC5C in the OE during regeneration of the OE. **A** Confocal images of OE cryosections of regenerating OE (3dpi, 14dpi and 28dpi), immunolabelled for GPRC5C (green) and GAP43 (red, left panel) and OMP (red, right panel). At 3dpi, no neurons were present and GPRC5C staining in the OE was not apparent. Dendritic knobs with GPRC5C expression can be seen at 14dpi and as regeneration of the neurons progresses, more knobs with GPRC5C staining are observed at 28dpi. **B** Quantification of GPRC5C positive knobs at 3-, 14- and 28-days post MMZ injection, counted in projections of confocal stacks of 16-μm thickness (*n* = 3 animals per group, individual data values Additional file 2, Student’s *t* test, error bars represent SEM, ***p < 0.001). **C** Confocal images of OE at 14dpi and 28dpi immunolabelled for GPRC5C (green) and γ-tubulin (red, left panel) and acetylated-tubulin (red, right panel). At 14dpi, GPRC5C + dendritic knobs have few newly formed acetylated-tubulin stained cilia, and at 28dpi, the amount of cilia increased. **D** Quantification of GPRC5C and γ-tubulin co-localization. Ca. 50% of the γ-tubulin + knobs express GPRC5C at 14dpi, at 28dpi ca. 80% of the dendritic knobs co-express GPRC5C and gamma-tubulin. Knobs were counted in projections of confocal stacks of 16-μm thickness, percentage co-expression was analysed using Student’s *t* test (*n* = 3 animals per group, individual data values Additional file 2, error bars represent SEM, **p < 0.01). **E** *Z*-stack projections of confocal images of WT and *Gprc5c*^−/−^ adult mouse OE cryosections showing comparable ARL13B staining. **F** Quantifications of staining intensity of ARL13B. Student’s *t* test showed no significant difference (*n* = 3, individual data values Additional file 2). Scale bars 10 µm

Owing to the localization of GPRC5C, we wanted to investigate its involvement in the formation of cilia. OSNs display cycles of birth, maturation and death due to ongoing neurogenesis. Formation of new neurons can be experimentally induced by severe injury to the OE by intraperintoneal injection of methimazole [39, 40]. To study GPRC5C expression and localization during the onset of ciliogenesis, WT animals were sacrificed at 3, 14 and 28 days post-injury (dpi), and analysed for the presence of GPRC5C. At 3dpi, neither neuronal markers nor GPRC5C expression was observed (Fig. 2A, B). Only few dendritic knobs with GPRC5C expression were observed at 14dpi, but the number increased during ongoing regeneration and regrowth of ciliary axonemes. GPRC5C was observed in dendritic knobs of both mature (OMP +) and immature (GAP43 +) neurons (Fig. 2A, B). Upon co-staining for GPRC5C and γ-tubulin, a marker for the ciliary basal body, we observed only about 50% of the knobs expressing γ-tubulin co-stain with GPRC5C (Fig. 2C, D). A significant increase in the percentage of knobs that co-expressed GPRC5C and γ-tubulin was observed at 28dpi (Fig. 2C, D). Acetylated-tubulin as marker for the ciliary axoneme showed few newly formed cilia at sites of GPRC5C-positive knobs at 14dpi (Fig. 2C). At 28dpi, GPRC5C and acetylated-tubulin labelling concomitantly increased. To summarize, during regeneration of the OE GPRC5C does not seem to be crucial for the formation of basal bodies, but is expressed concurrent with the appearance of acetylated-tubulin and may therefore play a role in olfactory cilia formation or maintenance.

Before the formation of multiple sensory cilia on their dendrites, immature OSNs build a primary cilium, which is required for proper maturation of OSNs and is distinct from the fully elongated cilia involved in odourant detection. The small GTPase ARL13B is expressed in these primary cilia, but is excluded from the cilia of mature OSNs [46]. Consistent with the absence of GPRC5C in primary cilia (Fig. 1H, I), ARL13B staining in dendritic knobs was not different in *Gprc5c*^−/−^ mice (Fig. 2E, F). Primary cilia with ARL13B are necessary for proper neuronal maturation [46]. Proper formation of ARL13B-positive primary cilia is well matched with the observation that absence of GPRC5C did not affect expression of proteins involved in neurogenesis, cell proliferation or apoptosis in the OE of young adult animals (Additional file 1: Fig. S3).

### Gprc5c knockout mice show alterations in ciliary morphology

**Fig. 3:**
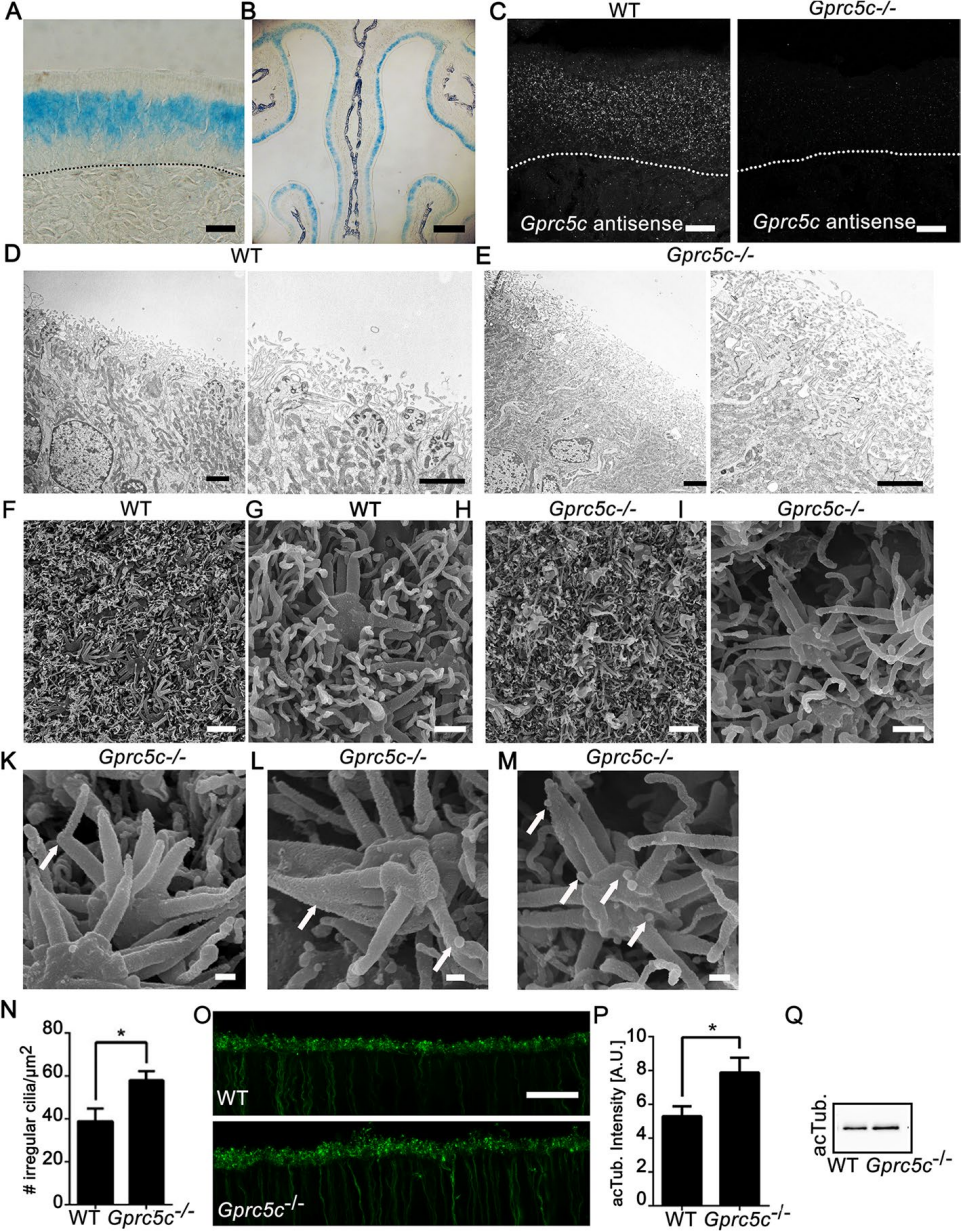
*Gprc5c*^−*/*−^ mice show altered ciliary morphology. **A** Bright-field image of ß-Gal staining of cryosections of *Gprc5c*^−/−^ adult mouse OE. ß-Gal staining is confined to the neuronal layer in the adult OE. Dotted line represents basal lamina. **B** Lower magnification overview image. **C** Confocal images of adult OE cryosection upon FISH of DIG-labelled probes for *Gprc5c* (antisense). *Gprc5c* mRNA was detected in WT, but not in *Gprc5c*^−/−^ OE. Dotted line represents basal lamina. Transmission electron micrographs of ultrathin sections OE of WT (**D**) and *Gprc5c*^−/−^ (**E**). *Gprc5c*^−/−^ OE depicts an increased thickness of the ciliary layer. **F** Scanning electron micrographs of WT OE appears organized with dendritic knobs evenly embedded in the surrounding ciliary network. **G** Higher magnification. **H** *Gprc5c*^−/−^ OE appears disorganized and has atypical cilia. **I** Higher magnification. **K** Cilium with bending of the distal end. **L** Clumped cilia. **M** Extracellular vesicles attached to cilia. **N** Quantification of irregular cilia. Mann–Whitney test showed significant increase in number of irregular cilia in *Gprc5c*^−/−^ OE (*n* = 4, individual data values Additional file 2, error bars represent SEM, *p < 0.05). **O** Staining of acetylated-tubulin (acTub) as marker for the axoneme of the cilia shows an increase in staining in *Gprc5c*^−/−^ OE. **P** Quantification of staining intensity followed by Student’s *t* test revealed a significant increase of acetylated-tubulin in *Gprc5c*^−/−^ OE (*n* = 4, individual data values Additional file 2, error bars represent SEM, *p < 0.05). **Q** Western blot analysis of whole OE preparations, acetylated-tubulin band at 52 kDa, levels were comparable between WT and *Gprc5c*^−/−^ OE, equal loading was controlled by actin. Scale bars **A**, **C**, **O** 10 µm; **B** 500 µm: **D**, **E**, **F**, **H** 2 μm; **G**, **I** 400 nm; **K**, **L**, **M** 200 nm

*Gprc5c*^−*/*−^ knockout mice were generated by inserting LacZ downstream of translation initiation site in exon 2 of *Gprc5c* [35], and 3-indolyl-ß-D- galactopyranoside X-gal staining showed blue-stained OSNs in all parts of the OE, as expected (Fig. 3A, B). No transcripts were detected in *Gprc5c*^−/−^ OE by in situ hybridization (Fig. 3C), and GPRC5C staining could not be detected in dendritic knobs (Additional file 1: Fig. S11). Interestingly, the total thickness of the ciliary layer appeared to be increased in *Gprc5c*^−/−^ OE (Fig. 3D, E). To investigate the morphology of the olfactory cilia in more detail in *Gprc5c*^−/−^ animals, we performed scanning electron microscopy. The dendritic knobs in WT OE were embedded uniformly amidst the surrounding meshwork of microvilli and cilia (Fig. 3F, G). The surface of the *Gprc5c*^−/−^ OE seemed to be less uniformly organized, and cilia showed atypical morphologies (Fig. 3H, I). The most prominently recurring irregularity in *Gprc5c*^−/−^ mice was the bending of the distal ends of the cilia at right angles (Fig. 3K). Occasionally, we observed clumping of the cilia at the proximal ends (Fig. 3L). We also encountered the presence of extracellular vesicles in the *Gprc5c*^−/−^ knockout OE (Fig. 3M), which may discard ciliary components to control of ciliary length or composition. Quantification of the number of atypical cilia indicated a significant increase the OE of *Gprc5c*^−/−^ animals (Fig. 3N). Also, staining for acetylated tubulin, a marker of the axoneme, reveals increased staining intensity in the ciliary layer of *Gprc5c*^−/−^ animals (Fig. 3O, P), consistent with a thicker ciliary layer, although overall expression level was comparable (Fig. 3Q).

### GPRC5C exhibits localization similar to ciliary gate proteins

**Fig. 4:**
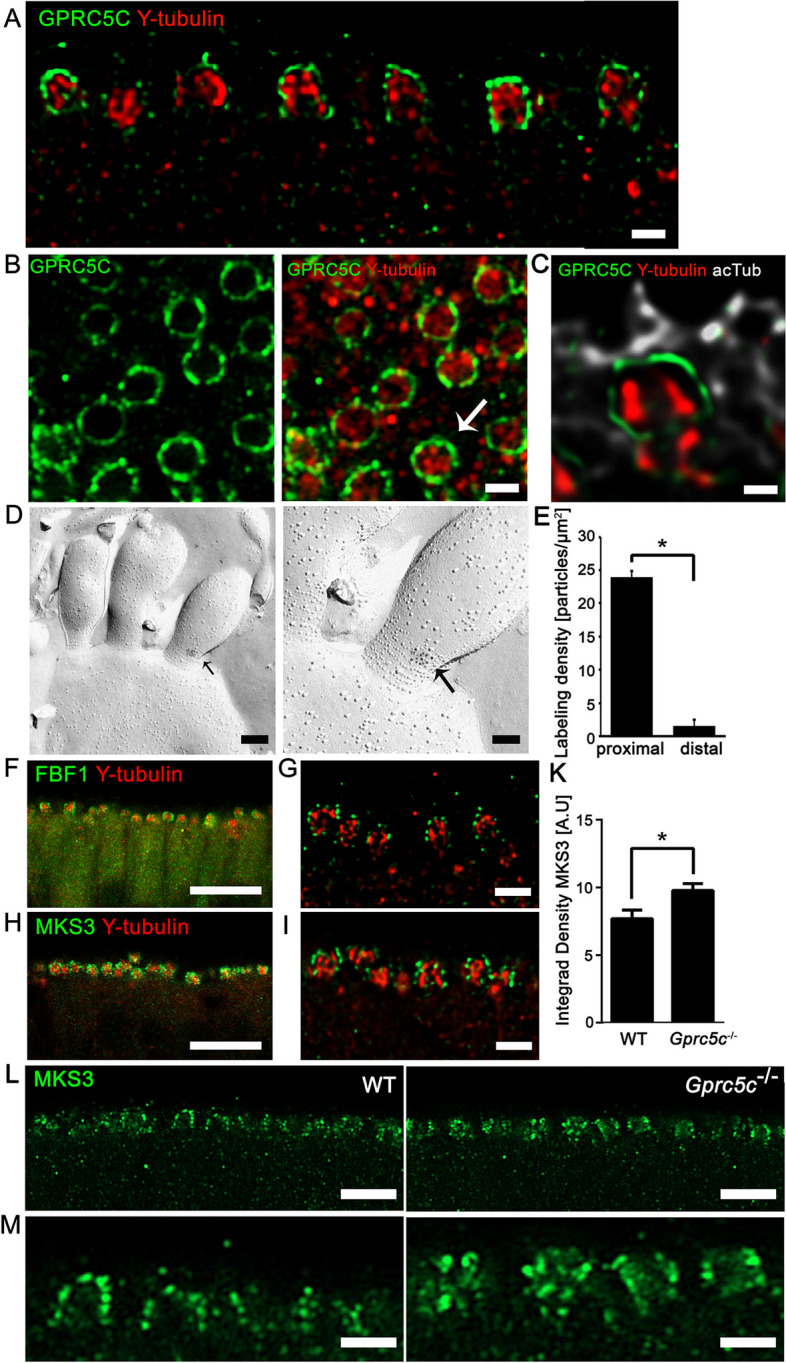
GPRC5C is localized at the ciliary gate. Confocal images of coronal sections (**A**) and en face preparation (**B**) adult mouse OE, immunolabelled for GPRC5C (green) and γ-tubulin (red) show punctate localization of GPRC5C along the membrane of the dendritic knob in a ring-like manner surrounding a γ-tubulin cluster that is localized in the lumen of the dendritic knob. Arrow indicates an individual dendritic knob. **C** Airyscan image with deconvolution processing of immunolabelling for GPRC5C (green), γ-tubulin (red) and acetylated-tubulin (white). GPRC5C is localized in the region between the basal body and the cilia. Arrow indicates an individual dendritic knob. **D** Immunogold labelling of freeze-fracture replica of the OE with GPRC5C antibody. Clusters of immunogold particles localized to the base of the cilia (black arrows). Higher magnification images show a distinct localization of GPRC5C at the ciliary necklace region, characterized by rows of ciliary proteins (arrow). **E** Quantitative analysis of anti-GPRC5C immunogold labellings of freeze-fractured plasma membranes of proximal and distal cilia. Proximal dendrite, *n* = 50 ROIs; distal dendrite, *n* = 40 ROIs. **F**, **G** Confocal images of FBF1 (green) and γ-tubulin (red), **H, I** MKS3 (green) and γ-tubulin (red). The localization pattern of FBF1 and MKS3 in the dendritic knob is similar to that of GPRC5C, all showing punctate staining along the membrane of the knob surrounding the γ-tubulin staining in the core. **K** Quantification of MKS3 staining intensity. Significance obtained upon performing Student’s *t* test (*n* = 3, individual data values Additional file 2, error bars represent SEM, *p < 0.05). **L, M** Confocal images of WT and *Gprc5c*^−/−^ OE immunolabelled MKS3 showing increased staining intensity and altered pattern of staining in *Gprc5c*^− −^dendritic knobs. Scale bars **A**, **B**, **G**, **I**, **M** 2 µm; **C** 1 µm; **D** 200 nm (right), 100 nm (left), **F**, **H** 10 µm; **L** 5 µm

GPRC5C staining was observed directly beneath the ciliary layer, but we did not find co-localization with the γ-tubulin positive basal bodies from which the axonemes of the cilia emanate. Individual dendritic knobs showed ring-like punctate arrangement of GPRC5C along the membrane, surrounding the central core of basal bodies marked by γ-tubulin (Fig. 4A, B). Using deconvolution processing of a high magnification image of an individual dendritic knob co-stained with GPRC5C, γ-tubulin and acetylated-tubulin, we observed that GPRC5C does not co-localize with either, but is instead localized in the region between the basal body and the proximal segment of the cilia (Fig. 4C). This region houses a specialized microdomain called the ciliary gate [47]. Immunogold labelling of freeze-fracture replica of the OE revealed localization of GPRC5C antibody at the necklace surrounding the base of the cilia (Fig. 4D), but rarely at the more distal end of cilia (Fig. 4E).

Having shown GPRC5C localization at the ciliary base, we compared the staining to Fas (TNFRSF6)-binding factor 1 (FBF1) as marker of the distal appendages [48], which play a role in membrane docking to initiate ciliogenesis. In addition, we localized Meckel-Gruber syndrome 3 (MKS3, also named transmembrane protein 67 (TMEM67) or Meckelin) at the TZ [48]. Upon co-staining with γ-tubulin, both FBF1 (Fig. 4F, G) and MKS3 (Fig. 4H, I) produced strikingly similar pattern of staining compared to GPRC5C. Both FBF1 and MKS3, showed punctate staining along the membrane of the dendritic knob, surrounding the cluster of γ-tubulin in the lumen. Unfortunately, we did not find antibodies suited for co-labelling these proteins with GPRC5C in the OE. We therefore next investigated if ciliary gate markers show altered expression in the *Gprc5c*^−/−^ OE. While immunofluorescence labelling for FBF1 showed no differences between WT and *Gprc5c*^−/−^ OE (Additional file 1: Fig. S4), MKS3 staining appeared to be more prominent in *Gprc5c*^−/−^ OE (Fig. 4K–M). Upon closer observation, we noticed that the individual puncta of MKS3 were bigger, brighter and unevenly distributed in *Gprc5c*^−/−^ dendritic knobs (Fig. 4M). Quantification of the intensity of MKS3 staining in individual dendritic knobs revealed a significant increase in staining intensity (Fig. 4K). No difference was observed for γ-tubulin staining of the basal body between WT and *Gprc5c*^−/−^ OE (Additional file 1: Fig. S4).

### Knock-out of Gprc5c causes altered localization of ciliary markers

**Fig. 5:**
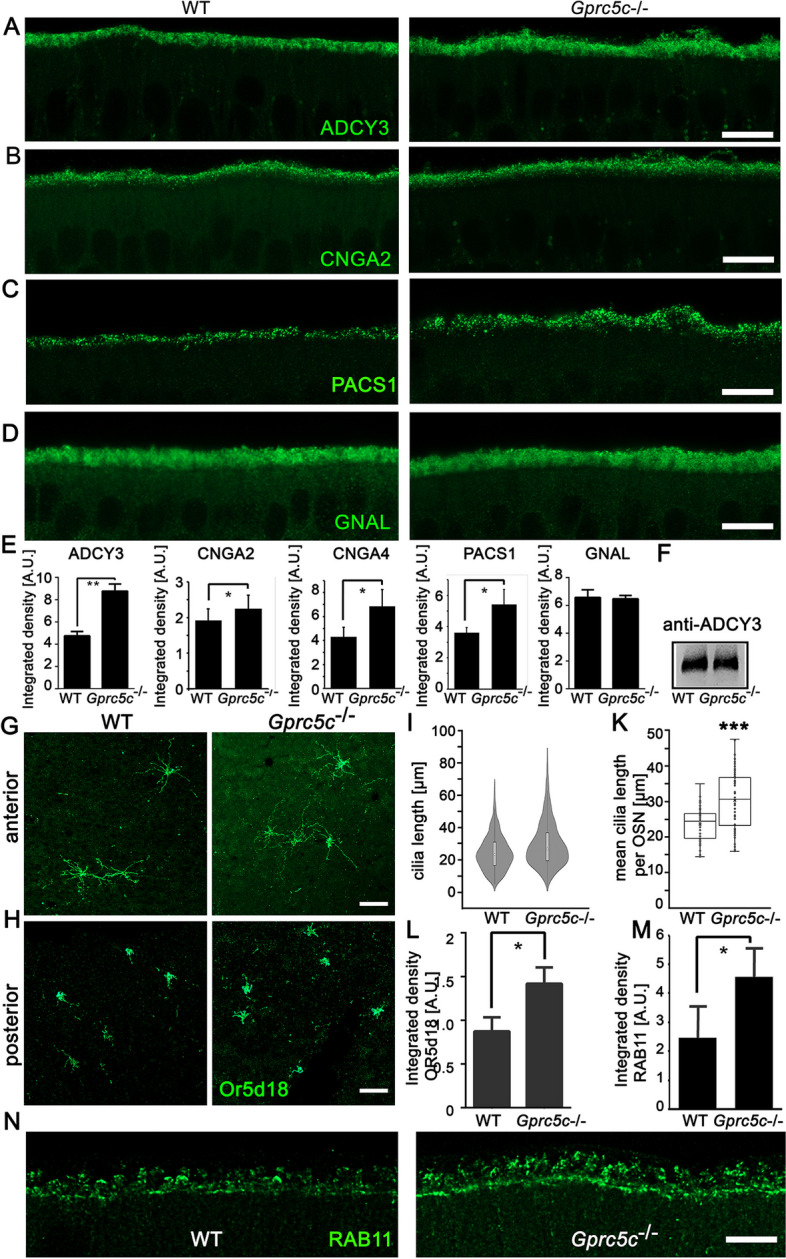
Localization of ciliary markers in WT and *Gprc5c*^−/−^ OE. *Z*-stack projections of confocal images of WT and *Gprc5c*^−/−^ adult mouse OE cryosections immunolabelled for **A** ADCY3, **B** CNGA2, **C** PACS1 and **D** GNAL. Scale bar 10 µm. While GNAL (Gαolf) staining is comparable between WT and *Gprc5c*^− −^ADCY3, CNGA2 and PACS1 ≥ is increased in *Gprc5c*^−/−^ cilia. **E** Quantifications of staining intensity of ciliary markers. Student’s *t* test showed significant increase in staining intensity for ADCY3, CNGA2, CNGA4, PACS1 and GNAL (*n* = 3–4, individual data values Additional file 2, error bars represent SEM, *p < 0.05, **p < 0.05). **F** Western blot analysis of ADCY3 expression in whole OE preparations, band slightly above 180 kDa, equal loading was controlled by actin. **G** Confocal images of en face preparation of septal OE of adult WT and *Gprc5c*^−/−^ mice immune-labelled for Or5d18, depicting the anterior region. Scale bar: 10 μm. **H** In the posterior region, cilia are in general shorter compared to the anterior region. **I** Violin plots of length of Or5d18 cilia in WT and *Gprc5c*^−/−^ mice. **K** Quantification of the length of Or5d18 + cilia per OSN. Student’s *t* test showed significant increase in *Gprc5c*^−/−^ OE (*n* ≥ 50 knobs, error bars represent SEM, ***p < 0.001). **L** Quantification of staining intensity of posterior mOR-EG neurons in WT and *Gprc5c*^−/−^ OE. Student’s *t* test showed significant increase in *Gprc5c*^−/−^ OE (*n* = 3, individual data values Additional file 2, error bars represent SEM, * *p* < 0.05). **M** Increased localization of RAB11-positive structures in the dendritic knobs of *Gprc5c*^−/−^ mice (*n* = 4 animals per group, individual data values Additional file 2, Student’s *t* test, error bars represent SEM, *p < 0.05). **N** *Z*-stack projections of confocal images of WT and *Gprc5c*^−/−^ OE cryosections immunolabelled for RAB11. Scale bar 10 µm

The ciliary gate is crucial for ciliogenesis and trafficking of proteins to the cilia as it acts as a docking site for IFT protein complexes [47]. Since we observed GPRC5C staining near the ciliary gate region, we investigated the localization of signalling proteins in cilia of WT and *Gprc5c*^−/−^ OE. Canonical odour response involves activation of Gαolf (GNAL) which in turn activates adenylyl cyclase type 3 (ADCY3) leading to cAMP-driven opening of cyclic nucleotide gated (CNG) channels (composed of CNGA2, CNGA4 and CNGB1). We observed an increase in ADCY3 staining in *Gprc5c*^−*/*−^ OE (Fig. 5A, E). The altered staining was likely caused by improper trafficking to the cilia, since the total protein level was not altered (Fig. 5F). Ciliary localization of CNGA2 and CNGA4, as well as phosphofurin acidic cluster-sorting protein 1 (PACS1), which plays a role in the ciliary trafficking of CNG channels [49], also increased (Fig. 5B, E). On the other hand, we did not observe differences in the ciliary expression of the chloride channel TMEM16B (data not shown). Moreover, Gαolf (GNAL), which associates to the membrane via palmitoylation, was comparable between WT and *Gprc5c*^−*/*−^ OE. Since we observed altered expression of different ciliary membrane proteins, we also investigated the localization of Or5d18, a well-characterized odourant receptor, in en face preparations of the septa WT and *Gprc5c*^−*/*−^ OE. We analysed the length of the cilia and found longer cilia in *Gprc5c*^−/−^ mice, as expected (Fig. 5I, K). We also observed distinct differences in ciliary length between Or5d18 expressing neurons depending on the anterior/posterior localization on the septum, as described previously [50]. In the posterior region with predominantly short cilia, the staining intensity was increased in *Gprc5c*^−*/*−^ mice (Fig. 5 G, H). In the anterior region with long cilia, we did not observe a significant difference in staining intensity between both genotypes, maybe due to a low signal to noise ratio of the thin ciliary structure extending over lager areas of the epithelium.

The small GTPase RAB11 at the base of the primary cilia is essential for protein entry into the cilium and cilia lengthening [51, 52]. RAB11 was localized in the dendritic knobs of OSNs, as well as in the apical cytoplasm of sustentacular cells to some degree (Fig. 5N). We quantified the staining intensities of RAB11 specifically in the dendritic knobs of WT and *Gprc5c*^−*/*−^ OE. Consistent with our observation of altered localization of membrane proteins and increased ciliary length, we detected enrichment of RAB11 in the dendritic knobs of *Gprc5c*^−*/*−^ mice (Fig. 5M, N).

### Absence of GPRC5C causes ciliary accumulation of PtdIns(4, 5)P2

**Fig. 6:**
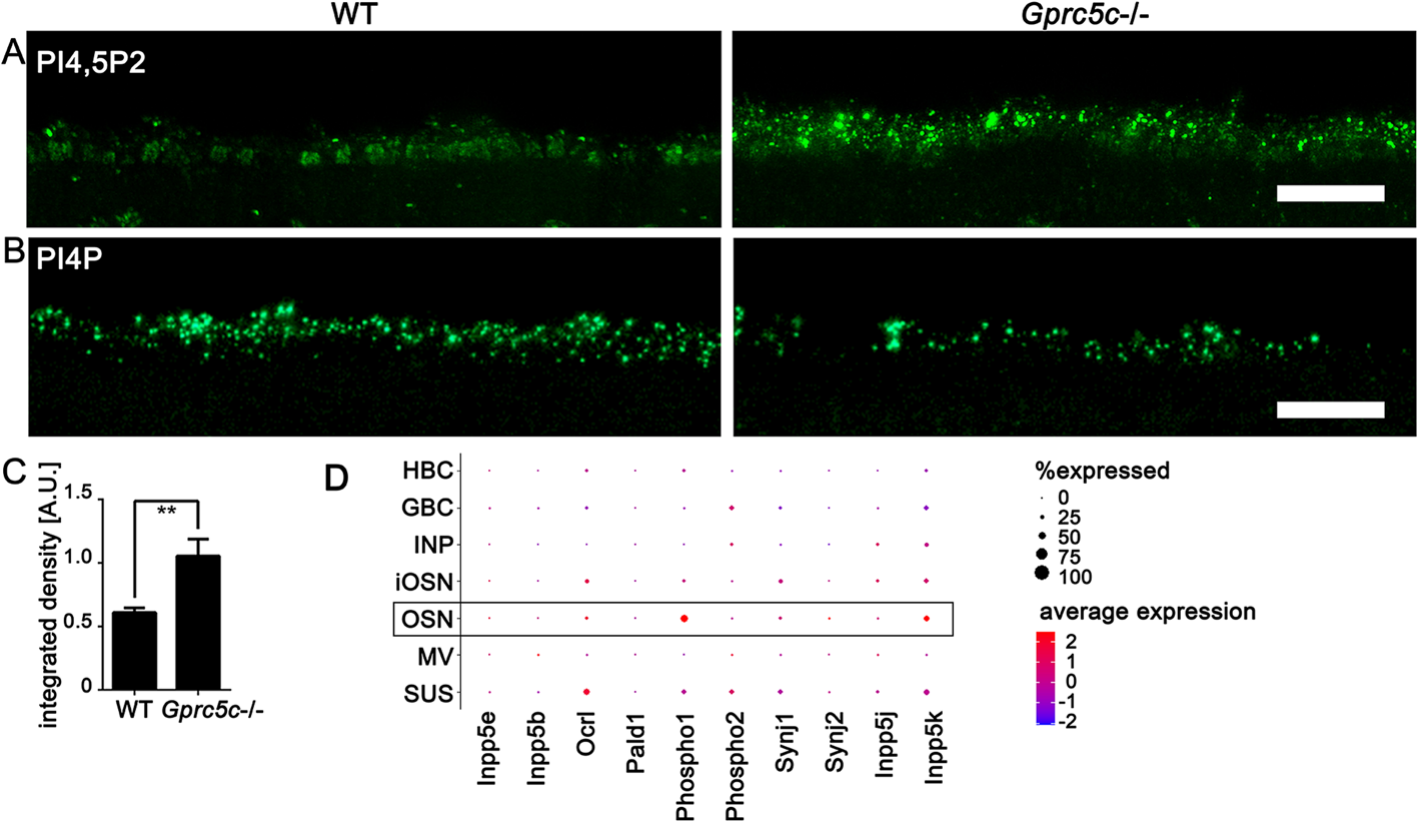
Redistribution of ciliary phospholipids in *Gprc5c*^−/−^ OE. Confocal images of WT and *Gprc5c*^−/−^ adult mouse OE cryosections immunolabelled for **A** PtdIns(4,5)P2 and **B** PtdIns4P. PtdIns(4,5)P2 staining is restricted to the dendritic knobs in WT OE whereas, *Gprc5c*^−/−^ OE shows several prominent puncta in the ciliary layer. PtdIns4P on the other hand showed a notable reduction in the *Gprc5c*^−/−^ cilia. **C** Quantification of PtdIns(4,5)P2 staining intensity. Student’s *t* test showed significant increase in staining intensity (*n* = 3, individual data values Additional file 2, error bars represent SEM, **p < 0.01). **D** Average expression and percent of respective OE cell types expressing different (putative) 5-phosphatases, OSNs are highlighted, data taken from [55]. HBC-horizontal basal cell, GBC-globose basal cell, INP-immediate neuronal progenitor cell, iOSN-immature olfactory neuron, OSN-mature olfactory neuron, MV-microvillar cell, SUS-sustentacular cell. Scale bars: **A, B** 10 µm

Phosphatidylinositol (4, 5)-bisphosphate (PtdIns(4, 5)P2) is localized at the base, but not along the axoneme of primary cilia and has been implicated in balancing membrane turnovers and ciliary disassembly through ciliary vesicle release by actin polymerization [53, 54]. PtdIns(4, 5)P2 has been shown to localize mostly in the dendritic knobs of OSNs and is only sparsely distributed in the cilia [11]. We confirmed this observation by immunofluorescence labelling of PtdIns (4, 5) P2 in WT mice. In sections of *Gprc5c*^−/−^ adult mice however, along with localization of PtdIns(4, 5)P2 in the knob, we also observed prominent staining in the ciliary layer (Fig. 6A, C). It is generally assumed that PtdIns (4, 5) P2 depletion in cilia is dependent on phosphoinositide 5-phosphatases [53], which hydrolyse PtdIns(4, 5)P2 to PtdIns4P. In line with the observation of increased PtdIns(4,5)P2 levels in cilia from *Gprc5c*^−/−^ mice, we found decreased PtdIns4P levels (Fig. 6B), indicating the decreased hydrolysis leads to alteration of PtdIns(4,5)P2 levels.

Conditional deletion of inositol polyphosphate-5-phosphatase E (INPP5E), which is causative of Joubert syndrome, leads to redistribution of PtdIns(4,5)P2 to the entire length of cilia and to a marked elongation of olfactory cilia [11]. Although GFP-INPP5E localizes to cilia in OE transduced with adenovirus encoding the GFP-INPP5E [11], staining with a commonly used INPP5E antibody did produce only a very faint labelling in the ciliary layer of the OE, together with marked unspecific labelling of the basolateral membrane (Additional file 1: Fig. S5). The ciliary staining seemed reduced in *Gprc5c*^−/−^ mice, but the non-specific labelling precludes unambiguous analysis of differences in the INPP5E staining. It is therefore unclear if INPP5E contributes to the observed effect on PtdIns(4,5)P2 levels in *Gprc5c*^−/−^ OE. To test if also other 5-phosphtases are expressed in OSNs, we surveyed a published single-cell olfactory transcriptome [55] and queried the dataset for expression abundance of several 5-phosphatases known to be involved in conversion of PtdIns(4,5)P2 to PtdIns4P (Fig. 6D). Specifically, we selected synaptojanin ½ (SYNJ1/2), INPP5E, INPP5J, oculocerebrorenal syndrome of Lowe (OCRL), INPP5B, and INPP5K, of which INPP5E/B and OCRL have already been shown to alter ciliary lipid composition [54, 56]. Moreover, recent proteomic analysis revealed that an important function of PALD1, which can release phosphate from PtdIns(4,5)P2, as attenuator of PtdIns(4,5)P2 hydrolysis-dependent Hedgehog signalling in cilia and phylogenetic pathway clustering analysis identified two more phosphatases, PHOSPHO1 and PHOSPHO2, co-conserved with PALD1 suggesting role for the local production of Pi in ciliary processes [57]. The transcriptome data show that *Ocrl*, *Phospho1* and *Inpp5k* are the most abundantly expressed genes (Fig. 6D), indicating that not only INPP5E, but also additional phosphatases may be involved in PtdIns(4,5)P2 hydrolysis in olfactory cilia. Moreover, other proteins without 5-phophatase activity such as AURKA and HDAC6 have been shown to affect PtdIns(4,5)P2 synthesis cilia [58, 59], leaving the underlying molecular mechanism of altered PtdIns(4,5)P2 distribution in *Gprc5c*^−*/*−^ mice unclear.

### Absence of GPRC5C causes increased neuronal activity and altered odour response

**Fig. 7:**
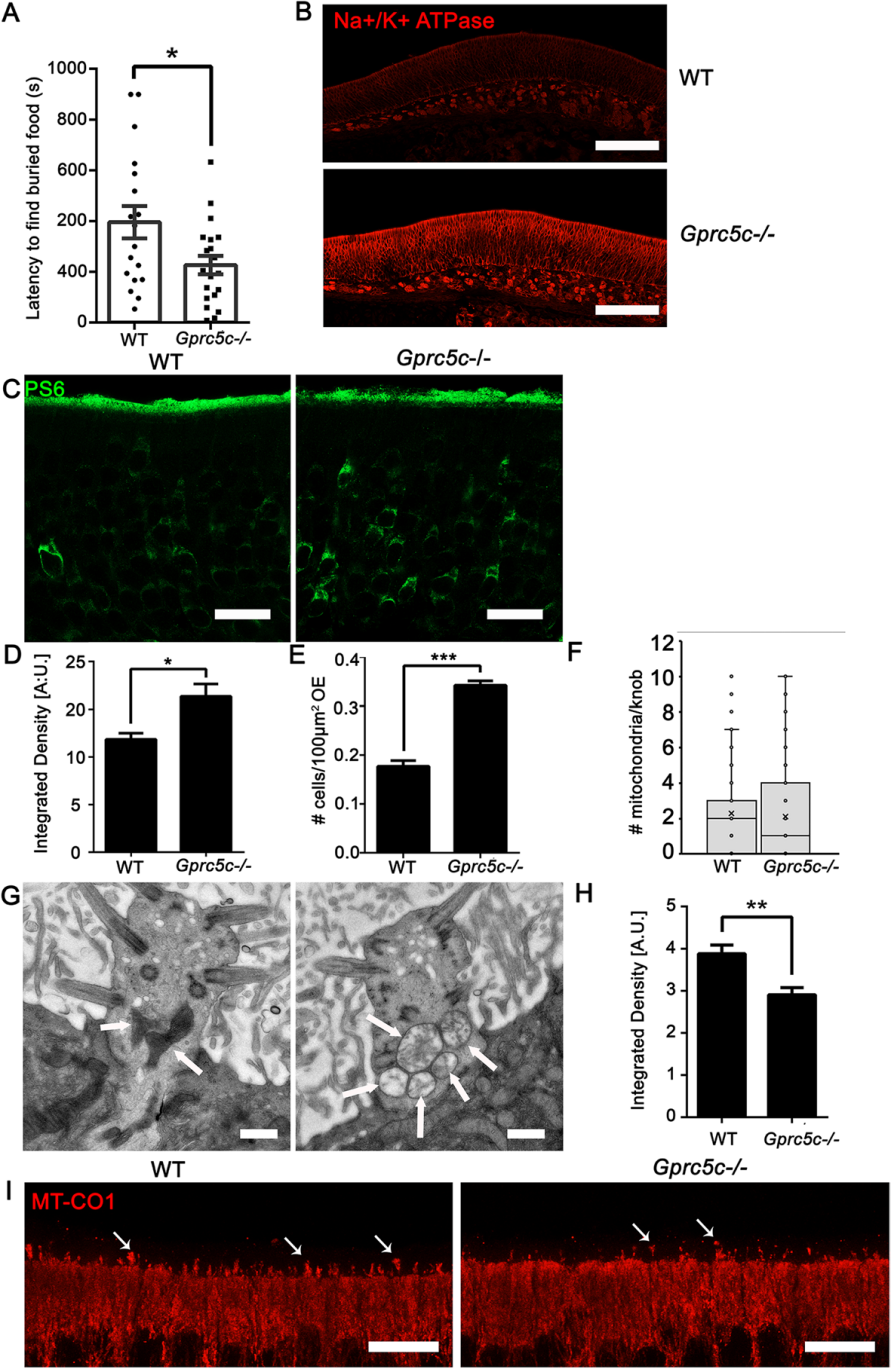
Effects of GPRC5C *knockout* on neuronal activity and behaviour. **A** Quantification for buried food test. *Gprc5c*^−/−^ mice exhibit a significantly reduced latency (WT 395.4 ± 63.66 s, *Gprc5c*^−/−^ 226.5 ± 36.49 s) to find buried food compared to WT mice, indicating an altered behavioural response (*n* = 18 animals per group, Student’s *t* test, error bars represent SEM, * *p* < 0.05).** B** Tile scans of the septum of the OE stained for Na/K + ATPase, both images were taken with the same microscope settings. **C** Z-stack projections of confocal images of WT and *Gprc5c*^−/−^ adult mouse OE cryosections immunolabelled for PS6. **D** Quantification of staining intensity of PS6 neurons in WT and *Gprc5c*^−/−^ OE Student’s *t* test showed significant increase in *Gprc5c*^−/−^ OE (*n* = 3, individual data values Additional file 2, error bars represent SEM, *p < 0.05).** E** Quantification of number of PS6 neurons. Student’s *t* test showed significant increase in *Gprc5c*^− −^E (*n* = 3, individual data values Additional file 2, error bars represent SEM, ***p < 0.001). **F** Quantification of the number of mitochondria per dendritic knob (*n* = 155 WT, *n* = 162 *Gprc5c*^−/−^). Mann–Whitney test shows no significant difference. **G** TEM of ultrathin sections of WT and *Gprc5c*^−/−^ OE depicting altered mitochondria in dendritic knobs of *Gprc5c*^−/−^ OE. Scale bar 400 nm. **H** Quantification of MT-CO1 staining as mitochondrial marker. Student’s *t* test shows significant reduction in staining intensity of MT-CO1 in dendritic knob layer of *Gprc5c*^−/−^ OE (*n* = 3, individual data values Additional file 2, error bars represent SEM, **p < 0.01). **I** *Z*-stack projections of confocal images of WT and *Gprc5c*^−/−^ adult mouse OE cryosections immunolabelled for MTCO1 (red) showing reduced staining in dendritic knobs of *Gprc5c*^−/−^ OE. Scale bar 10 μm

Considering the functions of the mis-localized ciliary proteins, we assumed that olfactory signalling is dysregulated in *Gprc5c*^−*/*−^ mice. We therefore investigated if the alterations in localization of ciliary proteins would translate to a behavioural change in *Gprc5c*^−*/*−^ animals. We performed the buried food/cookie finding test in which mice were starved overnight and the latency to find a piece of palatable cookie hidden below the bedding was monitored. *Gprc5c*^−*/*−^ mice exhibited a shorter latency to find the food compared to WT mice (Fig. 7A). This enhanced performance in olfaction may be linked to an increase in neuronal activity since we found increased staining of the Na^+^**-**K^+^ pump that controls the intrinsic activity mode of neurons (Fig. 7B). Moreover, immunofluorescence labelling for phosphorylation of serine 235/236 of ribosomal protein S6 (RPS6), known to be present in OSN in an activity-dependent manner [60], was increased in *Gprc5c*^−*/*−^ OE (7C-E). RPS6 is phosphorylated by p70S6kinase, which is a downstream target of mechanistic target of rapamycin complex 1 (mTORC1) [61]. Activation mTORC1 also results in autophagosome formation, and *Gprc5c*-deficient mice have multivesicular bodies in their dendritic knobs (Additional file 1: Fig. S6).

Neurons critically depend on mitochondria to establish membrane excitability and for neurotransmission and pathological neuronal activity results in mitochondrial and metabolic dysfunction. Transmission electron microscope (TEM) images of ultrathin sections of the OE showed that while to total number of mitochondria was not significantly different (Fig. 7F), many mitochondria in the dendritic knobs of *Gprc5c*^−*/*−^ OE displayed altered morphology (Fig. 7G). Immunofluorescence staining of mitochondrially encoded cytochrome c oxidase I (MT-CO1), which is a component of the respiratory chain, was reduced in dendritic knobs of *Gprc5c*^−*/*−^ compared WT OE (Fig. 7H, I). Because mitochondrial ultrastructure is determined by energetic demands, altered morphologies may be indicative of increased neuronal activity in OSNs.

## Discussion

Most eukaryotic cells contain microtubule-based organelles called cilia, which play a critical role in a number of biological processes. Significant structural heterogeneity exists among cilia, for example in terms of their length, proven by multiple mutations in ciliary genes leading to tissue-specific phenotypes. Cilia in OSNs are unique; for example, centriole amplification occurs in early progenitor cells, which continue to divide before evolving into OSNs [62]. We investigate here the function of GPRC5C, an orphan G protein-coupled receptor not implicated in cilia function so far. GPRC5C is localized at the transition zone of cilia extending from OSNs. Absence of *Gprc5c* does not cause typical ciliopathy-related phenotypes, but alters the ciliary transport of components involved in olfactory signal transduction and detection of olfactory cues. GPRC5C therefore provides an example of a protein that is involved in the function of olfactory, but not in all kinds of cilia.

### GPRC5C in sensory cilia of mature OSNs

GPRC5C is a 7-transmembrane receptor that we report to be highly expressed in the OE, and to a lower extent in other tissues. Here, we show that GPRC5C localizes to the base of olfactory cilia in the dendritic knobs of OSNs. Analysis of the regenerating OE after chemically induced injury showed that activation of stem cells during early regeneration does not seem to require GPRC5C. We observed that receptor expression was limited to dendritic knobs after basal body formation, when the first sensory cilia emerged, and that both GPRC5C and acetylated tubulin were concurrently increased. These findings, together with the finding that GPRC5C is localized at the ciliary gate, suggest that GPRC5C is involved in the formation and/or maintenance of sensory cilia.

However, GPRC5C is not localized in primary cilia in other OE cells, such as horizontal basal cells or immature olfactory neurons. GPRC5C has been localized at the apical membrane of proximal tubule cells in the kidney where it modulates systemic pH homeostasis [36], but we did not find specific localization of GPRC5C at the base of primary cilia in the proximal tubule. Although the receptor was not evidently localized to primary cilia, we cannot rule out its localization to primary cilia in other tissues. GPRC5C expression has also been reported in other cells which have primary cilia, such as dedifferentiated vascular smooth muscle cells [63, 64], or hematopoetic stem cells [37, 65], but the subcellular localization is not clearly identified in these cells. Taken together, it remains to be investigated whether ciliary base localization of GPRC5C is specific for the base of olfactory sensory cilia.

### GPRC5C regulates ciliary localization of membrane proteins

Using immunofluorescence microscopy and immunogold TEM we show that GPRC5C localizes in the TZ, which plays essential roles in the regulation of ciliary composition and function. As a result, the ciliary layer is thicker in *Gprc5c*^−*/*−^ mice. The TZ structure is composed of the Meckel-Gruber syndrome complex and the nephronophthisis complex. MKS3 (*Tmem67*) is a central player, causative in all three major ciliopathies, Meckel-Gruber syndrome, Nephronophthisis and Joubert-Syndrome [47]. Depletion of MKS3 leads to cilium elongation [66, 67], and aberrant cilium elongation underlies the pathogenesis of MKS3-linked ciliopathies [66]. Regulation of cilia length by GPRC5C may involve proper localization of TZ proteins, since *Gprc5c*^−*/*−^ OSNs exhibited MKS3 mislocalization. Depletion of MKS3 also leads to perturbed localization of membrane-associated protein ARL13B in primary cilia, but ARL13B is not expressed in the GPRC5C-positive sensory cilia of mature OSNs [46]. Moreover, mislocalization of ALR13B was not causative for the altered ciliary length in MKS3-deficient cells [66].

Consistent with the fact that the transition zone regulates the entry and retention of ciliary proteins [47, 68], we found altered localization of ciliary membrane proteins such as ADCY3, CNGA4 and odourant receptors in *Gprc5c*^−/−^ mice, but only non-significant effects on CNGA2 localization. It is well known that ciliary proteins have different targeting requirements. Impaired ciliary trafficking of both, ADCY3 and CNGA2, occurs in absence of centrin 2 (CETN2), where the ciliary transition zone and basal body function are disrupted [69]. Specific mislocalization of ADCY3 is caused by overexpression of the SUMO protease SENP2, and ADCY3 mutants with defective SUMOylation do not localize to cilia [70]. On the other hand, overexpression of a dominant-negative phosphofurin acidic cluster-sorting protein 1 (PACS-1) prevents CNGA2 localization, but does not affect ciliary localization of ADCY3 [71]. The localization of the lipid-attached protein Gαolf was not affected in *Gprc5c*^−/−^ mice, reflecting the well-known complexity in the mechanisms determining the specificity in targeting of lipid-anchored versus membrane proteins. Gαolf localization to cilia is regulated by Lipopolysaccharide-responsive and beige-like anchor protein (LRBA) [72] and centrosomal protein 290 (CEP290) [9], but neither of these proteins regulate membrane protein transport. Interestingly, N-acetyl-d-glucosamine binds to recombinantly expressed GPRC5C and induces an increase in intracellular calcium levels and β-arrestin recruitment [37]. N-acetyl-d-glucosamine is part of the bone marrow extracellular matrix component hyaluronic acid, which preserves dormancy of haemotopoietic stem cells through GPRC5C [37]. Although very speculative, the so far unclear molecular function of GPRC5C might involve trafficking or sorting of glycoproteins such as ADCY3, which is highly glycosylated [70, 73].

Genetic ablation of *Gprc5c* results in longer cilia and increased levels of ADCY3, a key protein in olfactory signal transduction. Intriguingly, absence of ADCY3 on the other hand results in shorter cilia and a dramatically altered the cilia pattern [50]. Despite the observation that ciliary localization of ADCY3 is not required for cilia growth [70], cAMP stimulates ciliary growth in cultured cells [74, 75], and altered cAMP concentrations may be directly or indirectly responsible for the effects on olfactory cilia length. Consistent with a role for cAMP in length regulation, mice lacking cAMP degrading phosphodiesterases (PDE) from OSNs (*Pde1c*^−/−^; *Pde4a*^−/−^) exhibit dramatically shorter cilia in the dorsal zone [76]. Based on the fact that odour-induced transient cAMP signalling does not modulate cilia pattern [50], basal cAMP levels are likely to affect the growth and maintenance of cilia [76].

Consistent with altered membrane protein localization, we found increased amounts of RAB11 in the dendritic knobs. RAB11, which regulates vesicular trafficking from the trans-Golgi network and recycling endosomes to the plasma membrane, is localized at the base of the primary cilia, and inhibition of RAB11 blocks ciliogenesis [51]. Reduced RAB11 activation causes defects in primary cilium elongation and membrane protein ciliary translocation [77, 78]. Interestingly, GPRC5C localization in proximal cilia and knobs of OSNs is similar to Stomatin L3, a member of the stomatin-domain family, which resides in a Rab11-positive vesicle pool and associates with sensory transduction molecules [79–81].The precise molecular mechanisms of Stomatin L3 are not fully elucidated, but may comprise modulation of ion channel activities, and possibly also modulation of GPRC5 or other components of the ciliary base.

### GPRC5C regulates phosphoinositide distribution

The abnormal accrual of transmembrane ciliary proteins in *Gprc5c*^−/−^ cilia may also lead to redistribution of PtdIns(4,5)P2. Although being continuous with the plasma membrane, the ciliary membrane possesses a unique composition of PIs. Due to ciliary localisation of the PI 5-phosphatase INPP5E, PtdIns4P is the dominant phosphoinositide along the ciliary membrane, whereas PtdIns(4,5)P2 is mainly limited to the proximal part of the cilium and the ciliary base [11, 82, 83]. Cilia from *Gprc5c*^−*/*−^ OSNs showed increased PtdIns(4,5)P2 and reduced PtdIns4P levels indicating decreased 5-phosphatase activity, but we did not find clear evidence for mislocalization of INPP5E. Although faint ciliary localization of endogenous INPP5E was detected using an established INPP5E antibody, unambiguous quantification was not possible since the antibody also produces marked basolateral staining. In line with the weak ciliary labelling, INPP5E does not seem to be very abundant in the transcriptome of OSNs [55]. Steady-state localization of INPPP5E on the ciliary membrane requires ARL13B [84], which is not localized to sensory cilia of OSNs (this work and [46]), but the ciliary targeting mechanisms of farnesylated INPP5E are quite complex [85]. In addition to INPP5E, multiple phosphoinositide 5-phosphatases with overlapping functions control ciliary phosphoinositide composition and could also be involved in PtdIns(4,5)P2 hydrolysis in OSNs. It is nevertheless interesting to note that animals with increased PtdIns(4,5)P2 levels in olfactory cilia exhibit a marked elongation of cilia accompanied by faster odour response kinetics [11].

### GPRC5C alters mitochondrial morphologies

Loss of GPRC5C disturbs quiescence and stemness of haemotopoietic stem cells. Cells without *Gprc5c* expression showed enrichment of ‘oxidative phosphorylation’ and ‘reactive oxygen species’ pathways in their mRNA, as well as an increased number of active mitochondria [37]. Moreover, an association between GPRC5C expression and alteration of the metabolic profile has recently been demonstrated for leukaemia cells [86]. We had similar observations with altered mitochondrial morphology and a reduction in MT-CO1 staining in dendritic knobs of *Gprc5c*^−/−^ OSNs, possibly caused by increased OSN activity. The decrease in oxidative phosphorylation capacity of the mitochondria by loss of MT-CO1, the mitochondrial-encoded core subunit of the cytochrome c oxidase, may indicate that *Gprc5c*^−/−^ OSNs may become more glycolytic. It has been shown that loss of MT-CO1 in liver mitochondria of aged mice results in a switch to glycolytic energy supply with increased ATP and lactate levels [87]. Since ATP diffusion from the knob would likely be too slow to fuel chemotransduction at distal sites of olfactory cilia, energy for chemotransduction is generated by glycolytic processing of glucose incorporated from the mucus [88]. Therefore, longer cilia might increase glycolytic energy production.

Interestingly, GPCR5C expression has also been related to mitochondria in another context, since expression is increased when Parkin is present, which detects and removes dysfunctional mitochondria [89]. Interaction with mitochondria may be relevant for ciliogenesis, since mitochondria have been found to localize to the ciliary basal bodies and the transition zone and to play a role in ciliogenesis [68, 90]. A phenotypic overlap between mitochondrial disorders and cilia-related abnormalities has been observed in nephronophthisis, craniofacial anomalies, microcephaly, and obesity [91–96]. Additionally, mitochondrial stress in astrocytes affects ciliary transcriptional programme and cilia structure, demonstrating a connection between mitochondria and cilia [97].

### Gprc5c − / − OSNs show increased activity levels

Changing cilia length in OSNs has intriguing functional implications. Here we report that GPRC5C regulates the activity of OSNs and odourant-stimulated olfactory behaviour, indicating that endogenous GPRC5C negatively regulates odourant-evoked intracellular signalling. *Gprc5c*^−/−^ mice showed increased expression of the sodium/potassium adenosine-triphosphatase (Na + /K + -ATPase, ATP1A1), indicating the necessity for increased ion transport across the plasma membrane due to augmented generation of action potentials. We also demonstrated OSN activity by augmented phosphorylation on the ribosomal protein S6 (S6RP), an established marker for OSN activity levels [60, 98]. Increased pS6RP levels in *Gprc5c*^−*/*−^ mice may result from unrestricted ADCY3 transport in absence of GPRC5C, since ciliary cAMP signalling increases levels of phosphorylated S6RP via the mTOR pathway [99]. As a coincidence, genetic ablation of ADCY3 dramatically alters the cilia pattern, suggesting ADCY3’s position is directly correlated with its length [50].

The fact that the ligand capture rate of a cilium is higher if length increases [100], and the observation that OSNs with longer cilia are more sensitive to odourants [50], could explain the enhanced performance of *Gprc5c*^−/−^ animals in our behavioural test. The buried food test could also be impaired by deficits in central olfactory processing, which is less likely due to the fact that *Gprc5c* expression was not found in the brain, except in the olfactory bulb and the cerebellum’s granular and molecular layers [35]. The cerebellum is involved in coordination of voluntary movements and the control of posture and balance, but also takes part in cognitive abilities and social interactions. However, mice with impaired function of Purkinje cells and granular cells of the cerebellum do not show any alteration in the latency to find buried food [101], and directed sniffing towards olfactory cues is not apparently altered in animals with cerebellar lesions [102]. In general, primary sensory functions do not seem to be affected in animals with cerebellar damage [103]. Considering in addition that *Gprc5c*^−/−^ mice did not display prominent behavioural defects [35], a primary effect in the OE seems most plausible. Contrary to GPRC5C, most proteins regulating olfactory signalling described so far enhance the transduction process. Exceptions are regulator of G protein signalling 2 (RGS2), a negative regulator of odourant-evoked intracellular signalling acting via ADCY3 [104] and Cilia- and Flagella-Associated Protein 69 (CFAP69) [105], slowing the response kinetics of electrophysiological responses.

## Conclusions

We propose that GPRC5C regulates two relevant biological processes in OSNs, namely, ciliary function and membrane trafficking events that influence or affect ciliary function. GPRC5C localizes to the transition zone of sensory olfactory cilia extending from mature OSNs and affects the localization of MKS3, another transition zone protein. In its absence, an aberrant accrual of the different ciliary proteins takes place, among them ADCY3, a key protein in olfactory signal transduction, and, consequently increased odourant-evoked signalling. This study additionally highlights the specificity of ciliary targeting mechanisms by showing GPRC5C functions in long, non-motile cilia of OSNs. Moreover, we discover a connection between cilia and mitochondria in OSNs.

### Supplementary Information

**Additional file 1: Figure S1. **Antibody validation of GRPC5C antibody. **Figure S2.** Supplemental data for Fig. 1. (A-D) GPRC5C expression during post-natal development; (E) GPRC5C is not associated with primary cilia in the kidney. **Figure S3.** Neurogenesis is not affected in Gprc5c-/- OSNs. **Figure S4.** Localization of dendritic knob markers in Gprc5c-/- mice. **Figure S5.** Cross-reactivity of INPP5E antibody with ATP1A1. **Figure S6.** Multivesicular bodies in dendritic knobs of Gprc5c-/- OE. **Figure S7.** Uncropped pictures of blots, imaged with a Western Blot Imager. **Table S1.** Antibodies used in this study.

**Additional file 2. **Supporting data values.

## Data Availability

All data generated or analysed during this study are included in this published article and its supplementary information files (Additional files 1 and 2).

## Abbreviations

OSN: Olfactory sensory neurons
OE: Olfactory epithelium
TF: Transition fibre
TZ: Transition zone
IFT: Intraflagellar transport
