# Supplementary material for: GPRC5C regulates the composition of cilia in the olfactory system
Source: BMC Biol. 2023 Dec 18;21:292. doi: 10.1186/s12915-023-01790-0 (PMC10729543; doi:10.1186/s12915-023-01790-0)
Supplement: Supplementary file 1 — Additional file 1: Figure S1. Antibody validation of GRPC5C antibody. Figure S2. Supplemental data for Fig. 1. (A-D) GPRC5C expression during post-natal development; (E) GPRC5C is not associated with primary cilia in the kidney. Figure S3. Neurogenesis is not affected in Gprc5c-/- OSNs. Figure S4. Localization of dendritic knob markers in Gprc5c-/- mice. Figure S5. Cross-reactivity of INPP5E antibody with ATP1A1. Figure S6. Multivesicular bodies in dendritic knobs of Gprc5c-/- OE. Figure S7. Uncropped pictures of blots, imaged with a Western Blot Imager. Table S1. Antibodies used in this study. [file 12915_2023_1790_MOESM1_ESM.pdf]

## Supplemental Figures

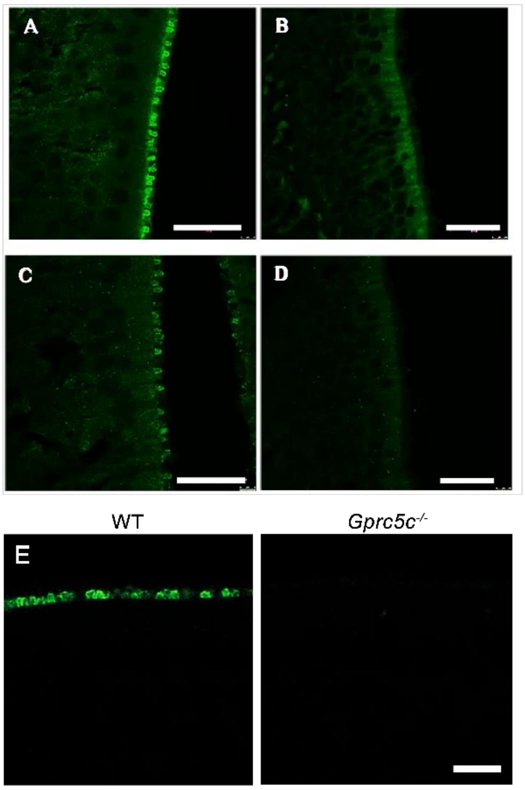


**Supplemental Figure S1 Antibody validation**

(**A**) Affinity chromatography purified anti-GPRC5C antibody showed staining specific to the dendritic knob layer of OSNs. (**B**) Secondary antibody control showed no staining (**C**) Faint staining of the knobs was also seen in sections treated with the immune serum. (**D**) No specific staining was seen in section treated with pre-immune serum. (**E**) Affinity chromatography purified anti-GPRC5C antibody showed staining in WT, but not in *Gprc5c-/-* OSNs. Scale bars 20μm

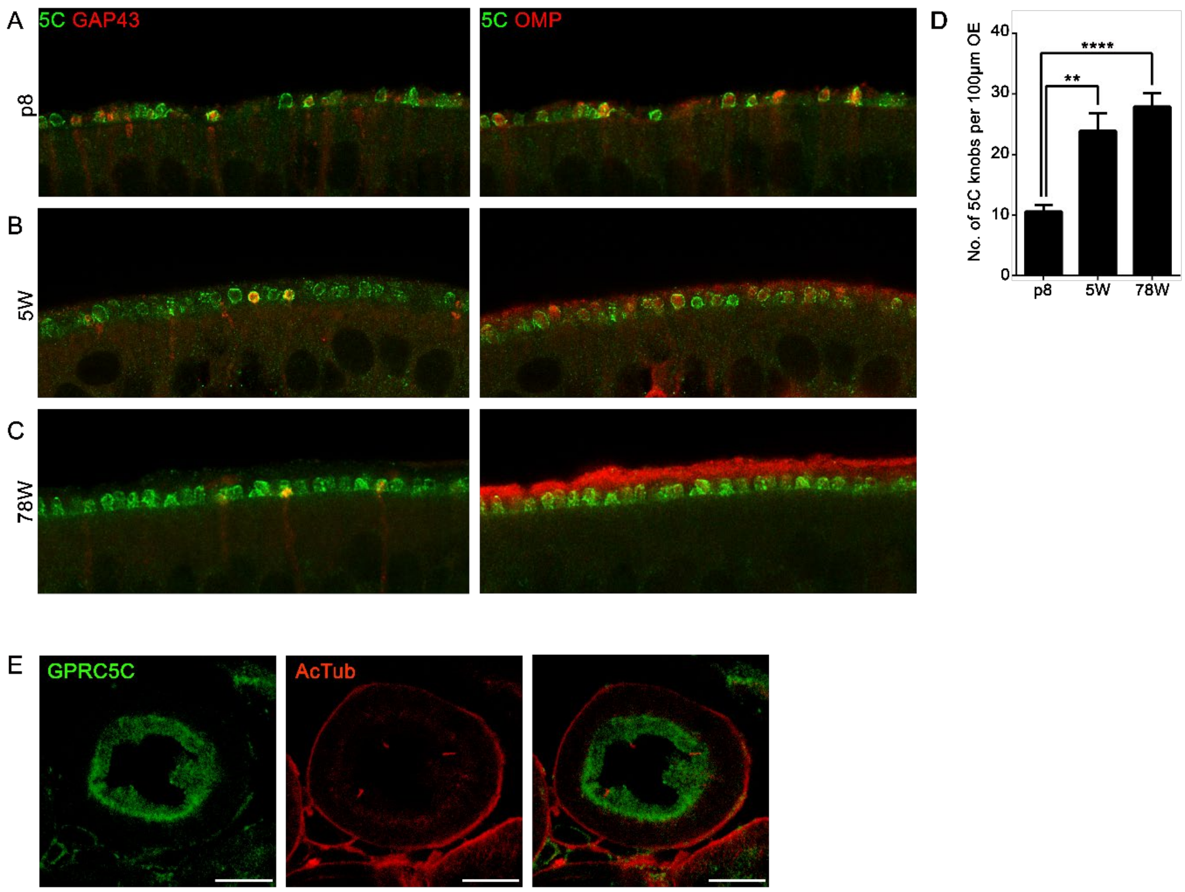


**Supplemental Figure S2. Supplemental data for Figure 1**

**(A-D) GPRC5C expression during post-natal development:** Confocal images of OE coronal cryosections of mice aged (**A**) 8 days postnatal (P8), (**B**) 5weeks (5W), and (**C**) 78 weeks (78W) immunolabeled for GPRC5C (green) and GAP43 (red) (left panel) and GPRC5C (green) and OMP (red) (right panel). GPRC5C staining is detected in the dendritic knobs of mature and immature neurons in young mice (P8) and continues to be expressed throughout adulthood (5W, 78W). (**D**) Quantification of GPRC5C positive knobs across different ages. One-way ANOVA and Tukey's multiple comparison test showed a significant increase in number of GPRC5C positive knobs in 5 weeks and 78 weeks old animals in comparison to p8 animals. (n=3, error bars represent SEM, ** $p < 0.05$, **** $p < 0.0001$). (**E**) **GPRC5C is not associated with primary cilia in the kidney:** Confocal images of single renal proximal tubule of mice aged 3 months immunostained with anti-GPRC5C antibody (green) and acetylated tubulin (red). Staining was not specifically associated with primary cilia. Scale bar 10µm.

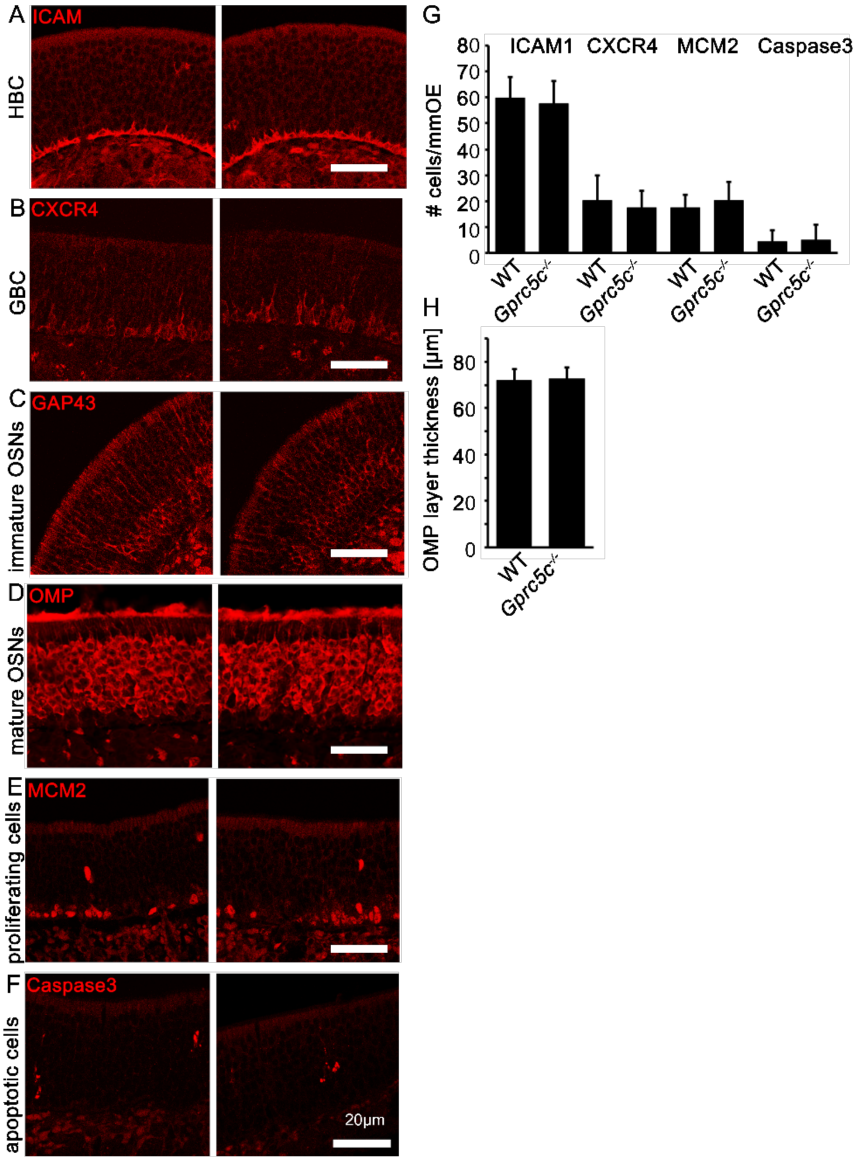


**Supplemental Figure S3. Neurogenesis is not affected in *Gprc*5c-/- OSNs**

Confocal images of WT and 5C-/- adult OE cryosections immunolabeled for: (**A**) ICAM1 as a marker for HBCs; (**B**) CXCR4 as a marker for GBCs; (**C**) GAP43 as a marker for immature OSNs; (**D**) OMP as a marker for mature OSNs; (**E**) MCM2 as a marker for proliferating cells and (**F**) Caspase3 as a marker for apoptotic cells. The labelling of these marker proteins is comparable between WT and *Gprc5c-/-* OE. (**G**) Cells counted in projections of confocal stacks of 16 µm thickness (n=3 animals per group, Student's t-test p>0.05, error bars represent SEM). (**H**) Thickness of the layer of OMP-positive mature neurons (n=3 animals per group, Student's t-test p>0.05, error bars represent SEM). Scale bars (A-F): 20 µm.

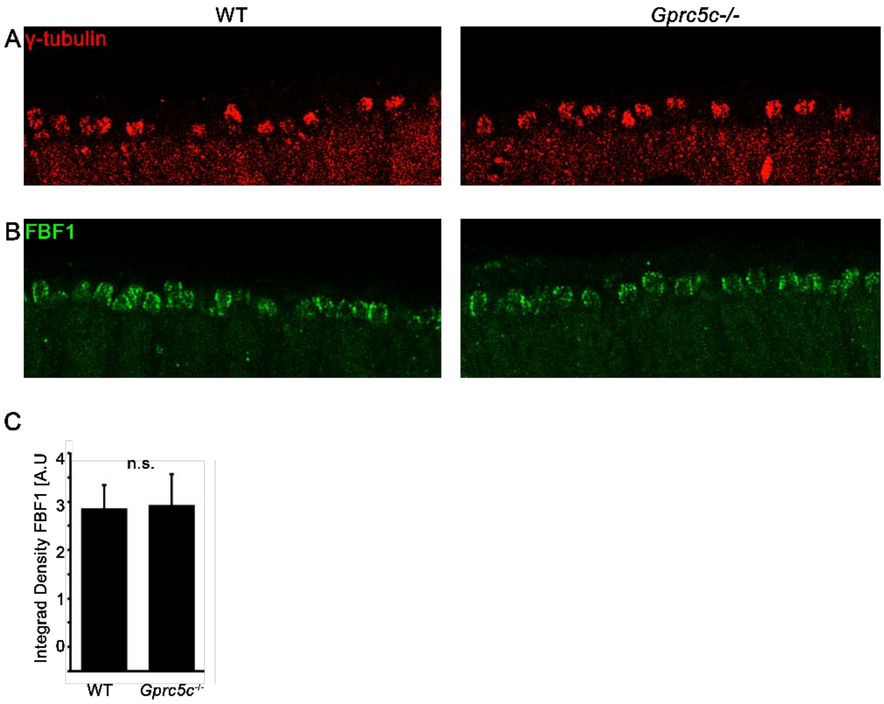


**Supplemental Figure S4. Localization of dendritic knob markers in *Gprc5c*-/- mice**

Confocal images of WT and *Gprc5c*-/- adult mouse OE cryosections immunolabeled for (**A**) γ-tubulin, a marker for basal bodies within dendritic knobs that is comparable between WT and *Gprc5c*-/- OE (**B**) Ciliary gate protein FBF1 showing no difference in staining pattern between WT and *Gprc5c*-/- OE. (**C**) Quantification of staining intensity of FBF1. Student's t-test showed no significant difference (n=3). Scale bar: 5 μm.

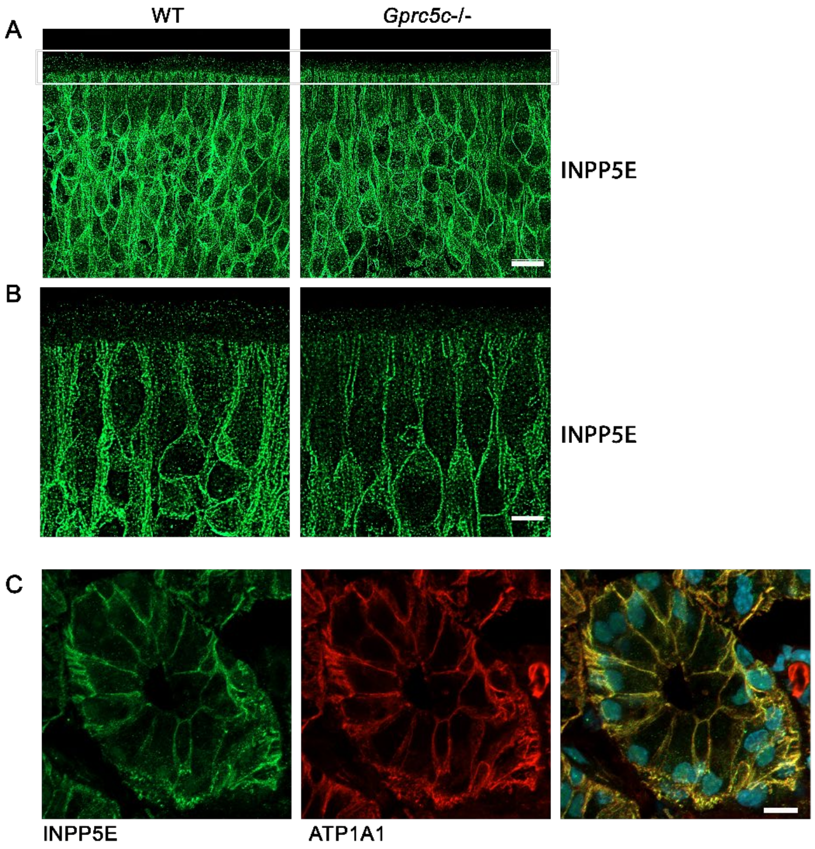


**Supplemental Figure S5. Cross-reactivity of INPP5E antibody with ATP1A1**

(A) Immunofluorescence labeling of cryosection of WT and Gprc5c-/- OE labeled with an antibody against INPP5E. Labeling occurs on the basolateral plasma membrane, only a very faint ciliary labeling (boxed) is present. Basolateral labeling may occur from labeling of ATP1A1, since both antibodies were reported to bind a shared epitope (https://zfin.org/ZDB-PUB-160713-11#fish). Scale bar 10µm. (B) Higher magnification. Scale bar 5µm. (C) Co-labeling of ATP1A1 (red) and INPP5E (green) in follicular cells of the thyroid gland, marked basolateral staining is present in both stainings indicating binding of the INPP5E antibody to ATP1A1. Scale bar 20µm.

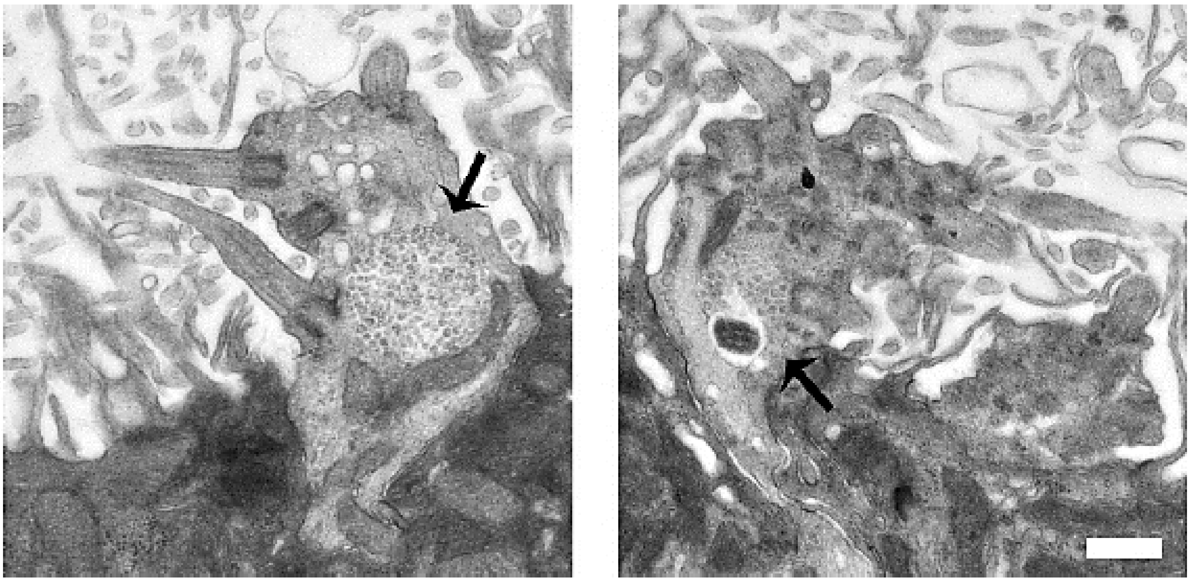

**Supplemental Figure S6 Multivesicular bodies in dendritic knobs of *Gprc5c-/-* OE** TEM analysis of ultrathin sections showing the presence of multivesicular bodies in the dendritic knobs of *Gprc5c-/-* OE. Multivesicular bodies are synonymous with late endosomes and belong to the endosomal pathway. Scale bar 200nm.

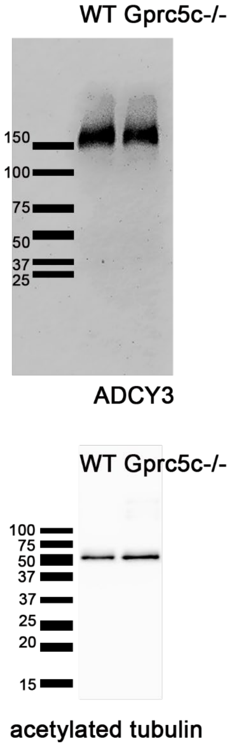


**Supplemental Figure S7 Uncropped pictures of blots, imaged with a Western Blot Imager.** Upper blot: anti-ADCY3 (Figure 5F); lower blot anti acetylated tubulin (Figure 3Q)

| Table S1: Antibodies used in this study. | | | |
|---|---|---|---|
| **antibody** | **host species** | **Dilution** | **company (order number)** |
| GPRC5C | rabbit | 1:200 | Home made (see Figure S1) |
| Acetylated tubulin | mouse | 1:1000 | Sigma (T-6793) |
| Phalloidin-A568 | | 1:500 | Invitrogen (A-12380) |
| IP3R | mouse | 1:200 | BD Biosciences (610312) |
| Arl13b | mouse | 1:200 | NeuroMab (N295B/66) |
| DCX | guinea pig | 1:100 | Synaptic Systems (326 004 LOT 1-4) |
| FBF1 | rabbit | 1:50 | Proteintech (11531-1-AP) |
| MKS3 | rabbit | 1:200 | BiCell Scientific (90103) |
| ADCY3 | rabbit | 1:200 | Santa Cruz (sc-588) |
| CNGA2 | goat | 1:200 | Santa Cruz (sc-13700) |
| CNGA4 | guinea pig | 1:100 | Michalakis S, Reisert J, Geiger H, Wetzel C, Zong X, Bradley J, Spehr M, Huttl S, Gerstner A, Pfeifer A, Hatt H, Yau KW and Biel M (2006). J Biol Chem 281:35156-66. doi: 10.1074/jbc.M606409200 |
| PACS1 | goat | 1:100 | Santa Cruz (sc-8465) |
| GNAL | rabbit | 1:200 | Santa Cruz (sc-383) |
| Or5d18 | rabbit | 1:200 | Home made (Rasche S, Toetter B, Adler J, Tschapek A, Doerner JF, Kurtenbach S, Hatt H, Meyer H, Warscheid B and Neuhaus EM (2010). Chem Senses 35:239-45. doi: 10.1093/chemse/bjq007 |
| PI4,5P2 | mouse | 1:200 | Invitrogen (A-21327, clone 2C11) |
| PI4P | mouse | 1:200 | Echelon Biosciences (Z-P004) |
| INPP5E | rabbit | 1:100 | Proteintech (17797-1-AP) |
| ATP1A1 | rabbit | 1:500 | Abcam (ab76020) |
| MT-CO1 | mouse | 1:100 | Abcam (ab14705) |
| Caspase3 | rabbit | 1:500 | Cell Signaling (9664S) |
| CXCR4 (2B11, N-term) | rat | 1:500 | Affymetrix (14-9991-82) |
| Gamma-tubulin | rabbit | 1:500 | Sigma (T3559) |
| GAP43 | mouse | 1:500 | Merck Millipore (MAB347) |

| | | | |
|---|---|---|---|
| MCM2 (BM-28) | mouse | 1:200 | BD Biosciences (610700) |
| ICAM1 | goat | 1:200 | R&D Systems (AF796) |
| OMP | goat | 1:500 | Wako (544-10001) |
| RAB11 | | 1:50 | Proteintech (CL488-15903) |
| pS6 | rabbit | 1:100 | Cell Signaling (2215S) |
| | | | |
| Alexa Fluor® 488 anti-goat | donkey | 1:500 | Thermo Fisher Scientific (A-11055) |
| Alexa Fluor® 488 anti-mouse | donkey | 1:500 | Thermo Fisher Scientific (A-21202) |
| Alexa Fluor® 488 anti-rabbit | donkey | 1:500 | Thermo Fisher Scientific (A-21206) |
| Alexa Fluor® 488 anti-rat | donkey | 1:500 | Thermo Fisher Scientific (A-21208) |
| Alexa Fluor® 568 anti-goat | donkey | 1:500 | Thermo Fisher Scientific (A-11057) |
| Alexa Fluor® 568 anti-mouse | donkey | 1:500 | Thermo Fisher Scientific (A-10037) |
| Alexa Fluor® 568 anti-rabbit | donkey | 1:500 | Thermo Fisher Scientific (A-10042) |
| Rhodamine Red ™-X anti-rat | donkey | 1:500 | Jackson Immuno Research (706-295-148) |
| Alexa Fluor® 633 anti-goat | donkey | 1:500 | Thermo Fisher Scientific (A-21094) |
| Alexa Fluor® 647 anti-rabbit | donkey | 1:500 | Thermo Fisher Scientific (A-31573) |
| Alexa Fluor® 647 anti-rat | donkey | 1:500 | Jackson Immuno Research (712-605-153) |
